# NLRP3‐Dependent Crosstalk between Pyroptotic Macrophage and Senescent Cell Orchestrates Trauma‐Induced Heterotopic Ossification During Aberrant Wound Healing

**DOI:** 10.1002/advs.202207383

**Published:** 2023-05-19

**Authors:** Juehong Li, Xin Wang, Zhixiao Yao, Feng Yuan, Hang Liu, Zhenyu Sun, Zhengqiang Yuan, Gang Luo, Xiangyun Yao, Haomin Cui, Bing Tu, Ziyang Sun, Cunyi Fan

**Affiliations:** ^1^ Department of Orthopaedic Surgery Shanghai Sixth People's Hospital Affiliated to Shanghai Jiao Tong University School of Medicine Shanghai 200233 China; ^2^ Shanghai Engineering Research Center for Orthopaedic Material Innovation and Tissue Regeneration Shanghai 201306 P. R. China; ^3^ Youth Science and Technology Innovation Studio of Shanghai Jiao Tong University School of Medicine Shanghai 200025 China

**Keywords:** cellular senescence, extracellular vesicles, heterotopic ossification, macrophages, pyroptosis

## Abstract

Heterotopic ossification (HO) represents an unwanted ossific wound healing response to the soft tissue injury which caused catastrophic limb dysfunction. Recent studies established the involvement of inflammation and cellular senescence in the tissue healing process, though their role in HO still remained to be clarified. Here, a novel crosstalk where the pyroptotic macrophages aroused tendon‐derived stem cells (TDSCs) senescence is revealed to encourage osteogenic healing during trauma‐induced HO formation. Macrophage pyroptosis blockade reduces the senescent cell burden and HO formation in NLRP3 knockout mice. Pyroptosis‐driven IL‐1*β* and extracellular vesicles (EVs) secretion from macrophages are determined to motivate TDSCs senescence and resultant osteogenesis. Mechanistically, pyroptosis in macrophages enhances the exosomal release of high mobility group protein 1 (HMGB1), which directly bounds TLR9 in TDSCs to trigger morbid signaling. NF‐*κ*B signaling is confirmed to be the converging downstream pathway of TDSCs in response to HMGB1‐containing EVs and IL‐1*β*. This study adds insights into aberrant regeneration‐based theory for HO formation and boosts therapeutic strategy development.

## Graphical abstract

After soft tissue injury, macrophages infiltrate the regenerative tissues and undergo pyroptosis, followed by the elevated release of IL‐1*β* and high mobility group protein 1‐containing extracellular vesicles. These pyroptotic macrophage‐driven paracrine factors, through toll‐like receptor 9 (TLR9)‐involved NF‐*κ*B signaling activation, intensify the senescent and osteogenic activity of tendon‐derived stem cells and thus encourage osteogenic healing of injured tissues, which eventually culminate in heterotopic ossification formation.

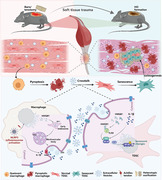

## 1 Introduction

Heterotopic ossification (HO) is characterized by the ectopic formation of bone within soft tissues, which often occurs after severe trauma incorporating complex fracture‐dislocation or could be regularly encountered after large surgery such as total joint arthroplasty or open reduction with internal fixation of elbow fractures.^[^ ^1^ ^]^ HO formation could compromise the range of motion of joints, causing functional loss of limb mobility and critically affecting patients’ daily activity. It has been reported that the incidence rate of HO could be as high as 36% and 15% after combat‐related injury and proximal humeral fractures, respectively.^[^ ^2^, ^3^ ^]^ However, current treatment options for HO and their success are still limited, making HO an intractable clinical challenge. Further in‐depth studies are thus urgently required to decipher the mechanism of HO formation and shed light on the development of new therapeutics.

Featured by the unexpected presence of bone within soft tissues, HO formation is essentially an aberrant osteochondral healing process in response to injury, though the rationale behind the abnormal healing tendency still remains to be clarified. Cellular senescence is characterized by growth arrest and phenotypic changes, which are typically found in natural aging but are recently suggested to be also induced after injury.^[^ ^4^, ^5^ ^]^ Scholars have proved that senescent cells could endanger tissue homeostasis or reshape the tissue healing process through endogenous functional reprogramming and the release of the senescence‐associated secretory phenotype (SASP).^[^ ^6^, ^7^, ^8^ ^]^ Especially, senescence of the mesenchymal stem cells (MSCs) that accumulated in response to injury dramatically impaired their intrinsic regenerative capacity and could lead to the erroneous cell fate commitment during differentiation, as exemplified in the osteoporotic lipogenic‐osteogenic imbalance.^[^ ^9^, ^10^ ^]^ An enlightening review summarized that patients of fibrodysplasia ossificans progressiva (FOP), a genetic form of HO, also shared some phenotypic features with those at premature aging state, indicating a possible involvement of cellular senescence in the HO progression.^[^ ^11^ ^]^ Accordingly, such a notion was subsequently corroborated in a transgenic mice‐based study conducted by wang et al.^[^ ^12^ ^]^ The results showed that senescent cell burden could be observed in the FOP, which imposed an osteochondral fate upon myogenic cells and thereby reprogramed tissue regeneration. However, the characterization and contributing factors of cellular senescence still need to be identified in trauma‐induced HO.

Inflammation has long been established to be one of the predominant stimulators for cellular senescence, especially for premature senescence.^[^ ^13^ ^]^ Macrophages constitute an important source of inflammation in innate immunity and thus may act as a potential culprit to cellular senescence induction. In our former study, we demonstrated that macrophages contributed to the pro‐inflammatory milieu after soft tissue injury and fueled the actuator of trauma‐induced HO formation.^[^ ^14^ ^]^ Pyroptosis is a form of programmed cell death that is frequently reported to be adopted by the macrophages to initiate inflammatory disorders.^[^ ^15^ ^]^ During this process, an inflammasome complex was formed to activate caspase and lead to N‐terminal domain liberation from the pore‐formation protein Gasdermin D. The resulting membrane pore formation, then caused the leakage of the intracellular components, including pro‐inflammatory cytokines and therefore incur dysfunction of surrounding cells in the milieu. Pieces of evidence of macrophage pyroptosis could be readily found in various injury‐associated conditions such as radiation‐induced lung fibrosis and ischemia stroke, wherein pyroptotic macrophages promoted pathological tissue remodeling.^[^ ^16^, ^17^ ^]^ Recently, pyroptotic macrophage was demonstrated to be capable of accelerating periodontal aging in diabetic periodontitis.^[^ ^18^ ^]^ Nevertheless, the role of macrophage pyroptosis and whether it gives rise to cellular senescence during trauma‐induced HO has not been elaborated.

In this study, we aimed to evaluate the effects of macrophage pyroptosis on cellular senescence and aberrant osteogenic healing during HO formation after soft tissue injury. Further, the underlying mechanism involved in the communication of pyroptotic macrophage with the affected senescent cells was elucidated. Our findings may provide novel insights into the pathological mechanism of trauma‐induced HO formation and inspire future therapeutic strategies.

## 2 Results

### 2.1 Pyroptotic Macrophages are Induced and Accompanied by Increased Cellular Senescence in the Trauma‐Induced Heterotopic Ossification

**Figure 1:**
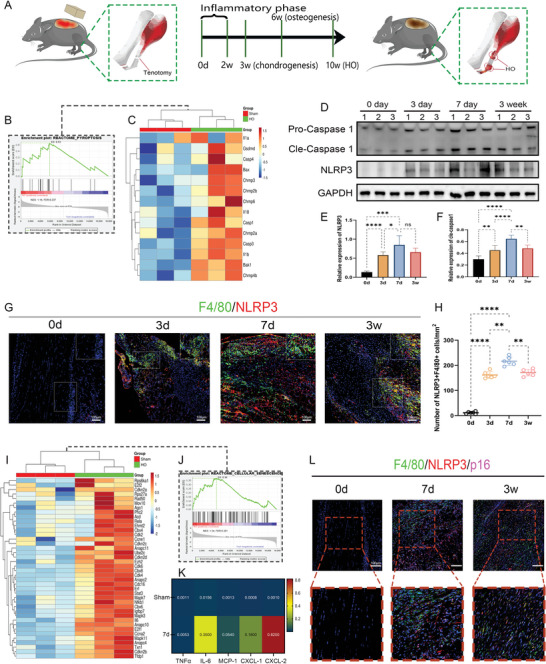
Macrophage pyroptosis occurred in accompany with cellular senescence during trauma‐induced heterotopic ossification (HO). A) Illustration of the murine modeling and representative developing stages for traumatic‐induced HO. B,C) Gene set enrichment analysis for the pyroptosis‐associated gene sets and heatmap for the expression level of representative pyroptosis‐associated genes in Sham (Control) and HO groups. D,E,F) Western blot (WB) analysis for pyroptosis‐associated protein expression, including NLRP3 and Caspase‐1, at different time points during HO formation. *N* = 3 per group. G,H) Double immunofluorescence staining of F4/80, NLRP3, and quantification of the number of NLRP3+F4/80+ cells at different time points during HO formation. *N* = 6 per group. I,J) Gene set enrichment analysis for the cellular senescence‐associated gene sets and heatmap for the expression level of representative cellular senescence‐associated genes s in Sham (Control) and HO groups. *N* = 3 per group. K) ELISA analysis for the senescence‐associated secretory phenotype (SASP) expression level, including TNF‐*α*, IL‐6, MCP‐1, CXCL‐1, and CXCL‐2. *N* = 3 per group. L) Triple immunofluorescence staining of F4/80, NLRP3, and p16 at different time points during HO formation. *N* = 6 per group. Data are presented as mean±SD. One‐way ANOVA followed by Tukey's test. *p<0.05, **p<0.01, ***p<0.001, and ****p<0.0001.

In order to determine the significant events involved in the trauma‐induced HO formation process, a murine burn/tenotomy model mimicking the trauma‐induced HO formation was established. The process of model establishment and different stage of HO development were depicted (**Figure** **1**A). RNA‐sequencing was performed with the lesion tissues collected at 3 weeks after injury, which localized at the central position of HO developing stages (Figure S1A–C, Supporting Information). Of particular importance to this study were the findings that gene expression related to pyroptosis was upregulated in the HO tissues, causing the high enrichment of the pyroptosis‐labeled gene in the GSEA analysis (Figure 1B,C). For further validation of the bioinformatic findings and more detailed exploration, characterization of the pyroptosis process was then carried out by Western blot (WB) analysis across different time points of HO evolution in the murine model. The results showed that the protein levels of pyroptosis‐related molecules incorporating NOD‐like receptor protein 3 (NLRP3), caspase‐1, and gasdermin D (GSDMD) were significantly enhanced during the early stage of the trauma‐induced HO. Furthermore, we found that the levels of pyroptosis peaked at 7 days after injury (Figure 1D–F). Since pyroptosis is a highly inflammation‐inducing phenomenon that is closely associated with the macrophages, we assumed that pyroptosis of macrophages happened during the HO formation. As expected, immunofluorescence staining revealed that macrophage marker F4/80 and pyroptosis marker NLRP3 both dramatically increased at 3 days, 7 days, and 3 weeks after the tendon injury, so did their colocalization, indicating the presence of macrophage pyroptosis during HO formation. In accordance with the WB results, the number of NLRP3+F4/80+ macrophages also reached the maximum at 7 days after injury, making day 7 an optimal time point for observation in the following experiments (Figure 1G,H). Next, further exploration of RNA sequencing data also indicated the distinct elevation of cellular senescence‐related gene expression in the HO group, which was reflected by the positive enrichment of these genes on the cellular senescence‐associated gene set in the GSEA analysis (Figure 1I,J). ELISA analysis for SASP also suggested the induction of cellular senescence during trauma‐induced HO formation. Anabatic release of several SASP‐associated cytokines incorporating IL‐1*β*, IL‐6, and MCP‐1 was found after the tendon (Figure 1K). Characterization of the cellular senescence was then performed in the murine model by staining for the senescence indicator p16, together with the F4/80 and NLRP3. The p16+ cells were observed to prominently accumulate in the injured tendon tissue, which was anatomically located near the pyroptotic macrophages (Figure 1L). These results implied the potential contribution of macrophage pyroptosis and cellular senescence to the HO evolvement and established the possible link between them. To specifically identify the cellular origin of these senescent cells, we checked the expression of specific tissue markers on p16+ cells through immunofluorescent staining. The results exhibited that p16 expression predominantly showed up in the PDGFR*α*+ osteoprogenitors and p16+ cells displayed a similar tissue distribution pattern with the PDGFR*α*+ osteoprogenitors, while the CD31+ endothelial cells, tenomodulin (TNMD)+ tenocytes, and *α*‐SMA+ fibroblasts appeared modest overlap with the p16+ cells. Little colocalization of p16 was observed with the F4/80+ macrophages (Figure S2A–E, Supporting Information). Considering the relatively pivotal role of tendon‐derived stem cells (TDSCs) as osteoprogenitors and direct contributors to tissue regeneration, we focused on the role of senescence of TDSCs and examined the rationale behind their senescence in the ensuing experiments.

### 2.2 Relief of Pyroptosis in NLRP3 Knockout Mice Attenuated the Senescence Cell Burden and HO Formation after Soft Tissue Trauma

**Figure 2:**
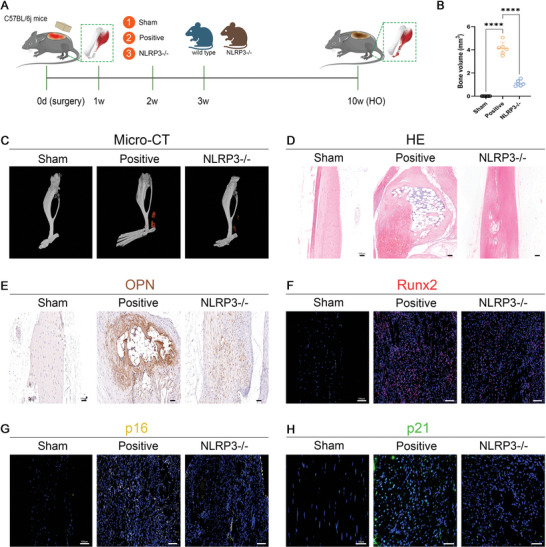
Quenching of pyroptosis diminished senescent cell burden and HO formation after tendon injury in the NLRP3 knockout (NLRP3−/−) mice. A) Illustration of the in vitro experimental treatment design. B,C) Micro‐CT analysis and quantification for HO formation in the wild type or NLRP3−/− mice at 10 weeks after burn/tenotomy. D) H&E staining of regenerative tendon tissues in the wild type or NLRP3−/− mice at 10 weeks after burn/tenotomy. E) Immunohistochemical staining of OPN in regenerative tendon tissues in the wild type or NLRP3−/− mice at 10 weeks after burn/tenotomy. F) Immunofluorescent staining of Runx2 in regenerative tendon tissues in the wild type or NLRP3−/− mice at 7 days after burn/tenotomy. G) Immunofluorescent staining of p16 in regenerative tendon tissues in the wild type or NLRP3−/− mice at 7 days after burn/tenotomy. H) Immunofluorescent staining of p21 in regenerative tendon tissues in the wild type or NLRP3−/− mice at 7 days after burn/tenotomy. *N* = 6 per group. Data are presented as mean±SD. One‐way ANOVA followed by Tukey's test. ****p<0.0001.

To elucidate the role of macrophage pyroptosis on the HO formation after injury, we employed NLRP3 general knockout (NLRP3−/−) mice which are defective for the key pyroptosis mediator protein (**Figure** **2**A). After burn/tenotomy injury, in comparison with the wild‐type mice, the NLRP3−/− mice showed sharp attenuation of the HO formation at 10 weeks as measured by the Micro‐CT, which were corroborated by the H&E staining results (Figure 2B–D). Immunofluorescent staining for Runx2 at 7 days and immunohistochemical staining for OPN at 10 weeks also demonstrated decreased osteogenic behavior in the NLRP3−/− mice (Figure 2E,F). Further, at 7 days after tendon injury, immunofluorescence staining for senescent markers p16 and p21 was performed to examine whether the activity of macrophage pyroptosis was correlated with the cellular senescence during HO formation. The results showed that the cellular senescence was also largely impeded after pyroptosis blocking in the NLRP3−/− mice, verifying the causal relationship between pyroptosis and cellular senescence in vivo (Figure 2G,H).

### 2.3 Pyroptotic Macrophages Contributed to the TDSCs Senescence and Aberrant Osteogenic Induction In Vitro

**Figure 3:**
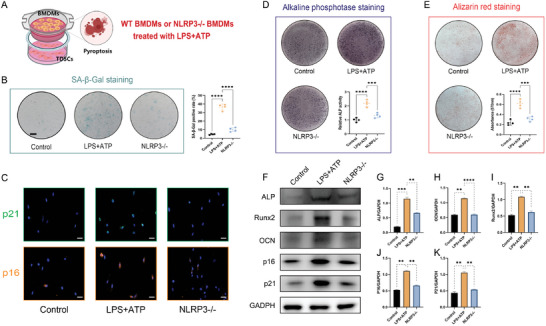
Macrophage pyroptosis contributed to the tendon‐derived stem cells (TDSCs) senescence and aberrant osteogenic activity. A) Illustration of the in vitro coculture system and experimental treatment design. B) Senescence associated *β*‐galactosidase (SA‐*β*‐Gal) staining for TDSCs after indicated treatments. C) Immunofluorescent staining for cellular senescence marker p16 and p21 in TDSCs after indicated treatments. D) Alkaline Phosphatase (ALP) staining for TDSCs following indicated treatments at 7 days after osteogenic induction. E) Alizarin red S (ARS) staining for TDSCs following indicated treatments at 21 days after osteogenic induction. *N* = 4 per group. F) WB analysis for cellular senescence marker p16, p21, and osteogenic marker Runx2, ALP, and OCN. G–K) Quantification of protein level of p16, p21, Runx2, ALP, and OCN normalized to GAPDH. *N* = 3 per group. Data are presented as mean±SD. One‐way ANOVA followed by Tukey's test. **p<0.01, ***p<0.001, and ****p<0.0001.

In light of that effects of macrophages were mainly realized with the help of their paracrine function, we explored whether the secretome from pyroptotic macrophages was responsible for the pro‐senescent effects on TDSCs. Coculture of the macrophages with the TDSCs was established using the Transwell system (**Figure** **3**A). Induction of macrophage pyroptosis was accomplished by stimulation with LPS followed by ATP. The results showed that after pyroptosis induction in macrophages, cellular senescence of TDSCs was dramatically enhanced, which was illustrated by the markedly increased SA‐*β*‐Gal staining proportion in TDSCs cultured with pyroptotic macrophages compared with that with unstimulated macrophages (Figure 3B). Similarly, the immunofluorescent staining exhibited enhanced p16 and p21 expression in the TDSCs after induction of macrophage pyroptosis. However, when the macrophage pyroptosis was blocked by NLRP3 deletion (using macrophages isolated from the NLRP3−/− mice), the senescence of the cocultured TDSCs showed significant attenuation (Figure 3C). In line with the changes in cellular senescence of TDSCs, the osteogenic potential of TDSCs was also heavily instigated after pyroptosis induction in cocultured macrophages but was largely ameliorated via the NLRP3 abrogation in the cocultured macrophages. To be specific, the evidence supporting the above‐mentioned statement was embodied in the alkaline phosphatase (ALP) and alizarin red S (ARS) staining as well as the WB analysis for the osteogenic protein marker involving ALP, Runx2, and osteocalcin (OCN) (Figure 3D–I). Impaired protein expression of p16 and p21 could also be observed in the TDSCs cocultured with macrophages isolated from the NLRP3−/− mice (Figure 3F,J,K). Besides, to directly establish the relationship between TDSCs senescence and its osteogenic activity, we employed hydrogen peroxide (H_2_O_2_) to induce TDSCs senescence and used quercetin to rejuvenate the senescent TDSCs due to its previous report as a senolytic drug for senescent stem cells elimination with high efficacy and low toxicity ^[^ ^19^ ^]^ (Figure S3A, Supporting Information). Before validation experiments, the cytotoxicity of quercetin was evaluated by CCK8 to exclude that the inhibitory effect of quercetin was not based on the cytotoxicity. The results showed that TDSCs kept normal proliferation under quercetin treatment for at least 7 days (Figure S3B, Supporting Information). Then, the SA‐*β*‐Gal staining along with the ALP and ARS staining testified that senescent TDSCs induced by H2O2 are equipped with higher osteogenic activity, whereas TDSCs rejuvenation by quercetin disabled its osteogenic behavior, indicating that TDSCs senescence leads to increased osteogenic differentiation (Figure S3C–H, Supporting Information). Since the secretome of pyroptotic macrophages could be primarily divided into the soluble fractions (conditioned medium without EVs) and EVs, we separately examined their roles in the behavior of TDSCs in the next experiments.

### 2.4 IL‐1*β* was Responsible for the Pro‐Senescent and Pro‐Osteogenic Effects of Pyroptotic Macrophages on TDSCs

**Figure 4:**
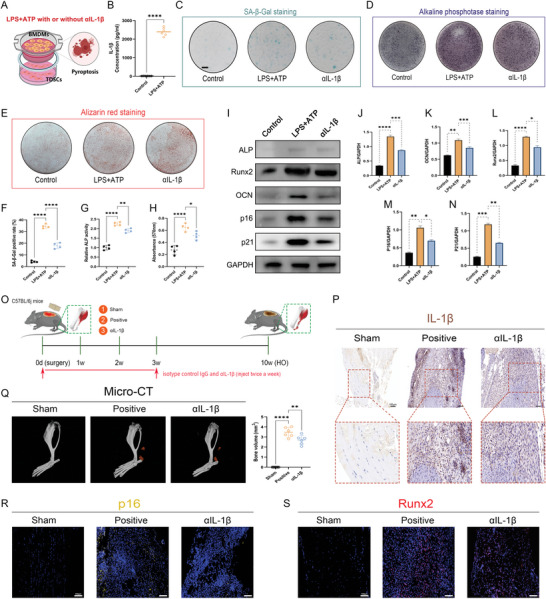
IL‐1*β* partially mediated the stimulating effects of pyroptotic macrophages on TDSCs senescence and osteogenesis. A) Illustration of the in vitro coculture system and experimental treatment design. B) ELISA analysis for the expression level of IL‐1*β* in the supernatants of macrophages after indicated treatments. C) SA‐*β*‐Gal staining for TDSCs after indicated treatments. D) ALP staining for TDSCs following indicated treatments at 7 days after osteogenic induction. E) ARS staining for TDSCs following indicated treatments at 21 days after osteogenic induction. F) Quantification of SA‐*β*‐Gal positive rate. G) Quantification of ALP activity for TDSCs following indicated treatments at 7 days after osteogenic induction. H) Quantification of absorbance of eluted ARS staining at 570 nm. *N* = 4 per group. I) WB analysis for cellular senescence marker p16, p21, and osteogenic marker Runx2, ALP, and OCN. J–N) Quantification of protein level of p16, p21, Runx2, ALP, and OCN normalized to GAPDH. *N* = 3 per group. O) Illustration of the experimental design in the murine burn/tenotomy model. P) Immunohistochemical staining of IL‐1*β* in regenerative tendon tissues following indicated treatments at 7 days after injury. Q) Micro‐CT analysis and quantification for HO formation following indicated treatments at 10 weeks after injury. R) Immunofluorescent staining of p16 in regenerative tendon tissues following indicated treatments at 7 days after injury. S) Immunofluorescent staining of Runx2 in regenerative tendon tissues following indicated treatments at 7 days after injury. *N* = 6 per group. Data are presented as mean±SD. One‐way ANOVA followed by Tukey's test. *p<0.05, **p<0.01, ***p<0.001, and ****p<0.0001.

The effects of the soluble fractions were first evaluated in vitro. Since IL‐1*β* has already been suggested as a potent inducer of cellular senescence during natural aging and meanwhile a featured cytokine released during pyroptosis, we testified whether IL‐1*β*accounted for the effects of soluble fractions on TDSCs. In the beginning, we checked the enrichment of IL‐1*β* in the supernatants of the pyroptotic macrophages (**Figure** **4**A). As expected, secretion of IL‐1*β* was dramatically increased after pyroptosis induction in macrophages (Figure 4B). In order to further elucidate the role of IL‐1*β*, rescue experiments were subsequently performed using the IL‐1*β* neutralization antibody. As suggested by the SA‐*β*‐Gal staining, the addition of the IL‐1*β* antibody significantly decreased the expression of *β*‐galactosidase in TDSCs (Figure 4C,F). In parallel, ALP and ARS staining suggested that the addition of the IL‐1*β* antibody also significantly abolished the osteogenic activity of TDSCs that was reinforced through coculturation with the pyroptotic macrophages (Figure 4D,E,G,H). WB analysis again verified the diminished existence of cellular senescence and osteogenic potential in TDSCs (Figure 4I–N). Moreover, the synergistic effects of IL‐1*β* with other proinflammatory cytokines that coexisted in the HO milieu were explored. As shown by the SA‐*β*‐Gal staining, ALP staining and ARS staining, TNF‐*α*, IL‐6, and TGF‐*β*1 separately owned the ability to facilitate TDSCs senescence and osteogenesis, while IL‐1*β* further reinforced their pro‐senescent and pro‐osteogenic effects on the TDSCs. These results revealed that IL‐1*β* could also play a supporting role in the senescence‐ and osteogenesis‐inducing mechanism of the inflammatory microenvironment within HO (Figure S4A–C, Supporting Information). Next, using the murine burn/tenotomy model, we also verified that IL‐1*β* expression could be raised by trauma in vivo (Figure 4O,P). Further, we also administrated the IL‐1*β* antibody to the murine traumatic‐induced HO model for evaluation of the effects of IL‐1*β* blocking. The results showed that Micro‐CT analysis at 10 weeks appeared damaged osteogenic healing after IL‐1*β* blocking, though the effects were not markedly (Figure 4Q). Accompanying this change, immunofluorescent staining for p16 was mitigated in the IL‐1*β* blocking group compared with that in the Sham group, alongside the reduced runx2 staining density, indicating a decreased load of senescent cells and osteogenic cells (Figure 4R,S). These results suggested that IL‐1*β* blocking could have an inhibitory effect on HO formation.

### 2.5 Extracellular Vesicles (EVs) Derived from the Pyroptotic Macrophages Contributed to the Senescent and Osteogenic Behavior of TDSCs During Trauma‐Induced HO Formation

For assessment of the role of extracellular vesicles from pyroptotic macrophages in trauma‐induced HO formation, GW4869 was applied to restrain the secretion of EVs. Results from the coculture assay showed that GW4869 supplementation reversed the pro‐senescent and pro‐osteogenic effects of pyroptotic macrophages on TDSCs (Figure S5A–M, Supporting Information) and the ultimate HO formation in the murine trauma‐induced HO model (Figure S5N–Q, Supporting Information).

**Figure 5:**
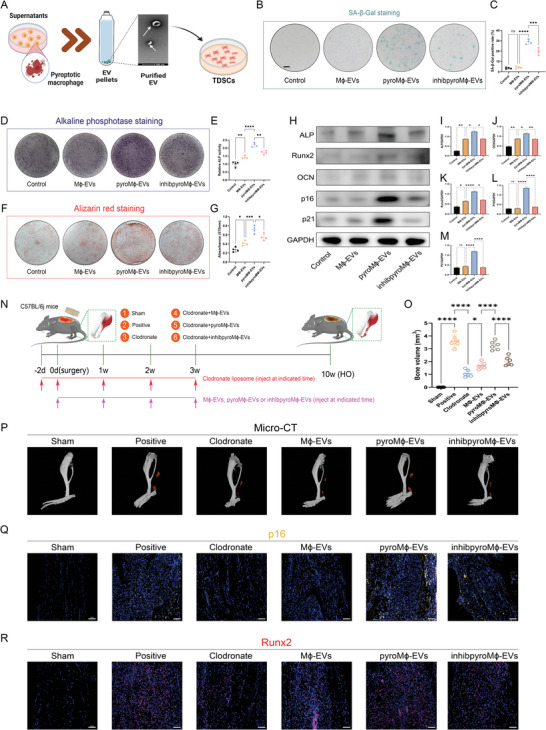
Extracellular vesicles (EVs) from pyroptotic macrophages triggered senescence and osteogenic induction of TDSCs. A) Illustration of the in vitro coculture system and experimental treatment design. B) SA‐*β*‐Gal staining for TDSCs after indicated treatments. C) Quantification of SA‐*β*‐Gal positive rate. D) ALP staining for TDSCs following indicated treatments at 7 days after osteogenic induction. E) Quantification of ALP activity for TDSCs following indicated treatments at 7 days after osteogenic induction. F) ARS staining for TDSCs following indicated treatments at 21 days after osteogenic induction. G) Quantification of absorbance of eluted ARS staining at 570 nm. *N* = 4 per group. H) WB analysis for cellular senescence marker p16, p21, and osteogenic marker Runx2, ALP, and OCN. I–M) Quantification of protein level of p16, p21, Runx2, ALP, and OCN normalized to GAPDH. *N* = 3 per group. N) Illustration of the experimental design in the murine burn/tenotomy model. O,P) Micro‐CT analysis and quantification for HO formation following indicated treatments at 10 weeks after injury. Q) Immunofluorescent staining of p16 in regenerative tendon tissues following indicated treatments at 7 days after injury. R) Immunofluorescent staining of Runx2 in regenerative tendon tissues following indicated treatments at 7 days after injury. *N* = 6 per group. Data are presented as mean±SD. One‐way ANOVA followed by Tukey's test. *p<0.05, **p<0.01, ***p<0.001, ****p<0.0001 and NS: not significant.

To better elaborate the action of the EVs, we directly isolated the EVs from the supernatants of unstimulated macrophages (M*ϕ*‐EVs), pyroptotic macrophages (pyroM*ϕ*‐EVs), or pyroptotic NLRP3−/− macrophages (inhibpyroM*ϕ*‐EVs) (Figure S6A, Supporting Information). Characterization of the isolated EVs was accomplished using transmission electron microscopy (TEM), nanoparticle tracking analysis (NTA), and WB analysis. Membrane‐bound cup‐shaped nanoparticles could be captured by the TEM, which conformed to the typical morphology of EVs (Figure S6B, Supporting Information). NTA determined the particle size of isolated EVs to be within the range of 30–150 nm (Figure S6C, Supporting Information). WB validated the abundant expression of the EVs markers incorporating CD9, CD63, CD81, ALIX, TSG101, and their parental cell marker F4/80, while the expression of the endoplasmic reticulum marker Calnexin was barely, collectively demonstrating the successful isolation of macrophage‐derived EVs (Figure S6D, Supporting Information). The uptake of EVs by TDSCs was also validated. Dil‐labeled EVs showed localization in the intracellular space of TDSCs, indicating excellent internalization, regardless of the origin of EVs (Figure S6E, Supporting Information). In line with what was found with GW4869, direct administration of pyroM*ϕ*‐EVs resulted in the elevated senescent cell abundance of TDSCs in the coculture system, which failed to be reproduced in inhibpyroM*ϕ*‐EVs (**Figure** **5**A–C). Osteogenic changes of TDSCs were slightly reinforced upon M*ϕ*‐EVs treatment, while intensely reinforced upon pyroM*ϕ*‐EVs treatment. However, the aforementioned pro‐osteogenic effects are pale upon inhibpyroM*ϕ*‐EVs treatment in comparison to the pyroM*ϕ*‐EVs (Figure 5D–G). Such osteogenic and senescent behaviors change was further confirmed by the WB analysis of related markers (Figure 5H–M). Consistent with this, the application of pyroM*ϕ*‐EVs boosted the HO formation in the murine trauma‐induced HO model in vivo, according to the results proposed by micro‐CT (Figure 5N–P). It was also shown that pyroM*ϕ*‐EVs resulted in higher production of senescent cells and greater pro‐osteogenic activity, manifesting as the enhanced expression of p16 and runx2 in pyroM*ϕ*‐EVs treated tissues compared to the normal tendon (Figure 5Q,R). Moreover, the in vivo fluorescent tracing experiments of EVs demonstrated that pyroM*ϕ*‐EVs could be absorbed by TDSCs and directly instigated TDSCs senescence, shown by the colocalization of PDGFR*α* and p16 with the DM‐Dil‐labeled EVs in the regenerated tendon. More particularly, the PDGFR*α*+ TDSCs that absorbed more pyroM*ϕ*‐EVs in the tissues also showed higher expression of senescent marker p16 than PDGFR*α*+ TDSCs that did not absorb pyroM*ϕ*‐EVs (Figure S7A,B, Supporting Information). Thus, we could conclude that either EVs or soluble fractions were indispensable for the full realization of the function of pyroptotic macrophages on TDSCs.

### 2.6 EVs from Pyroptotic Macrophages Shuttled High Mobility Group Protein 1 (HMGB1) to Incur Senescent and Osteogenic Changes of TDSCs After Soft Tissue Trauma

**Figure 6:**
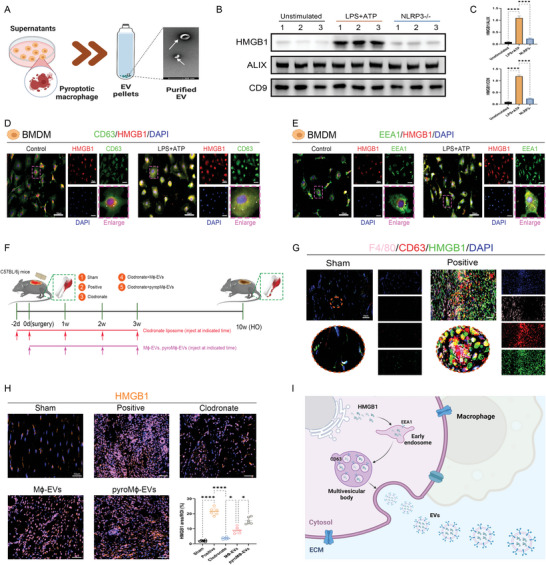
EVs from pyroptotic macrophages shuttled HMGB1 for HO modulation. A) Illustration of in vitro experimental protocols. B) WB analysis for HMGB1 protein expression in EVs after indicated treatments and C) quantification of HMGB1 relative protein level that normalized to ALIX and CD9. *N* = 3 per group. D) Double immunofluorescence staining for CD63 and HMGB1 in BMDMs after pyroptosis induction. E) Double immunofluorescence staining for EEA1 and HMGB1 in BMDMs after pyroptosis induction. F) Illustration of the experimental design in the murine burn/tenotomy model. G) Triple immunofluorescence staining for CD63, HMGB1, and F4/80 in the regenerative tendon at 7 days after injury. Round images in the lower panel are magnified images of the circled area in the upper panel. H) Immunofluorescence staining of HMGB1 in regenerative tendon tissues after indicated treatments at 7 days after injury and Quantification of the fraction of HMGB1 area to the area of a range of interests (ROI). *N* = 6 per group. I) Schematic diagram of incorporation of HMGB1 into the EVs for trafficking upon macrophage pyroptosis. Data are presented as mean±SD. One‐way ANOVA followed by Tukey's test. *p<0.05, ***p<0.001, and ****p<0.0001.

Then, a question that remained to be answered is what content mediated the biological effects of EVs. HMGB1 has been regarded as one of the most powerful damage‐associated molecular patterns (DAMPs) that are only released during macrophage pyroptosis due to the lack of signal peptide and are supposed to be possibly encapsulated into vesicles.^[^ ^20^ ^]^ Therefore, we checked the yields of HMGB1 in the pyroM*ϕ*‐EVs (**Figure** **6**A). WB analysis proposed that compared to the unstimulated macrophages‐derived EVs, more HMGB1 was generated in the pyroM*ϕ*‐EVs, whereas HMGB1 encapsulation was significantly restrained in the EVs derived from pyroptosis induced NLRP3−/− macrophages (inhibpyroM*ϕ*‐EVs) (Figure 6B,C). For an explanation of the incorporation of HMGB1 into pyroM*ϕ*‐EVs, immunofluorescence for the HMGB1 was performed in the bone marrow‐derived Macrophages (BMDMs) with or without pyroptosis induction. It could be observed that upon pyroptosis induction, more HMGB1 translocated from the nucleus to the cytoplasm of macrophages alongside the speckle‐like distribution of the endosome system marker CD63 and EEA1. Further, the cytoplasmic HMGB1 showed colocalization with these CD63+ or EEA1+ speckles, indicating the loading of the HMGB1 into the endosome system for EVs formation and extracellular release in response to the pyroptosis induction (Figure 6D,E). To validate these findings in vivo, triple immunofluorescent staining for F4/80, CD63, and HMGB1 was also performed in the regenerative tendon tissues 7 days after burn/tenotomy (Figure 6F). Compared with the Sham‐operated tendon, the regenerative tendon revealed a dramatic elevation of the expression of CD63 and HMGB1 in addition to the increased extracellular distribution of the HMGB1, accompanied by the ample infiltration of the F4/80+ macrophages. In terms of the histological location, within the Positive group, HMGB1 colocalized with the CD63, and such colocalization was found to be situated around the F4/80+ cells concentrated areas, suggesting the macrophage responsible for releasing the HMGB1‐containing vesicles (Figure 6G). Next, for more direct confirmation of the increased HMGB1 trafficking by the pyroM*ϕ*‐EVs during the trauma‐induced HO formation, we applied clodronate for macrophage depletion and directly injected EVs. The efficiency of macrophage depletion and EVs incorporation was illustrated in Figures S8A–C and S9A,B (Supporting Information). Immunofluorescent staining for HMGB1 examination showed that HMGB1 expression was substantially augmented following a tendon injury and redistributed to the extra‐nuclear region, which, however, was largely impeded by macrophage elimination. Nevertheless, when M*ϕ*‐EVs or pyroM*ϕ*‐EVs were added, the diminished HMGB1 could be rescued to a high level (Figure 6H). Therefore, the above results indicated that pyroptotic macrophages could secrete EVs to shuttle HMGB1 and contribute to trauma‐induced HO formation. The generation and packaging process of HMGB1‐containing EVs is summarized (Figure 6I).

Subsequently, for better determination of the role of HMGB1 involved in the EVs, we performed HMGB1 gene overexpression and knockdown in the macrophages through transfection (Figure S10A–E, Supporting Information). Analysis of the contents of the isolated EVs demonstrated anticipant upregulation or downregulation of HMGB1 protein enrichment after corresponding transfection and pyroptosis induction (Figure S10F,G, Supporting Information).

As for functional identification of the acquired HMGB1‐overexpressed pyroM*ϕ*‐EVs (OV‐EVs) or knockdown pyroM*ϕ*‐EVs (KD‐EVs), in vitro osteogenic induction of TDSCs was conducted. The results confirmed that OV‐EVs but not KD‐EVs successfully gave rise to the cellular senescence of TDSCs and was more potent than pyroM*ϕ*‐EVs, which led to more SA‐*β*‐Gal staining as well as more protein production of senescence marker p16 and p21 in TDSCs (Figure S11A–C,H,L,M, Supporting Information). Regarding osteogenic changes, OV‐EVs strengthened the osteogenic commitment of TDSCs and were even superior to the pyroM*ϕ*‐EVs, as revealed by the more intensified ALP and ARS staining combined with the expression of osteogenic marker protein, whereas KD‐EVs failed to recapitulate the pro‐osteogenic activity of pyroM*ϕ*‐EVs (Figure S11D–G,H–K, Supporting Information).

After that, further specification of the function of HMGB1 was performed in vivo by application of the OV‐EVs or KD‐EVs following GW4869 pretreatment to create the EVs‐depleted situation. According to the micro‐CT results, mice that received the KD‐EVs developed less HO than that received pyroM*ϕ*‐EVs, whereas adoption of the pyroM*ϕ*‐EVs and OV‐EVs aggravated the HO formation that was inhibited by GW4869 after tendon injury, with OV‐EVs aggravated more ( Figure S11N–P, Supporting Information). Parallelly, administration of KD‐EVs hardly reversed the inhibitory effects of GW4869 on cellular senescence and osteogenic potential, in contrast to results obtained from pyroM*ϕ*‐EVs and OV‐EVs, wherein OV‐EVs exerted more pro‐senescent and osteogenic effects than pyroM*ϕ*‐EVs (Figure S11Q,R, Supporting Information). In view of these findings, it could be concluded that pyroptotic macrophages could secrete the HMGB1‐enriched EVs to promote the senescent and osteogenic process during the HO formation.

### 2.7 HMGB1 in Pyroptotic Macrophage‐Derived EVs Exerted Pathogenic Effects through Binding to TLR9 in TDSCs

**Figure 7:**
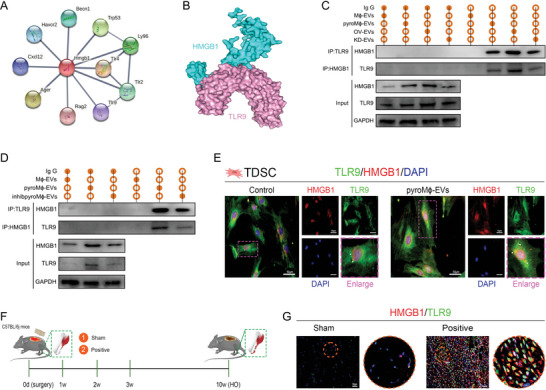
Exosomal HMGB1 bound TLR9 to modify aberrant behaviors of TDSCs during traumatic HO formation. A) Protein–protein interaction network for HMGB1 established by STRING database. B) Molecular docking of murine HMGB1 and TLR9. C,D) Coimmunoprecipitation (Co‐ip) analysis for binding between HMGB1 and TLR9 in TDSCs after indicated treatments. *N* = 3 per group. E) Double immunofluorescence staining of HMGB1 and TLR9 in TDSCs. *N* = 4 per group. F) Illustration of murine burn/tenotomy modeling. G) Double immunofluorescence staining of HMGB1 and TLR9 in regenerative tendon tissues at 7 days after injury. *N* = 6 per group.

To further decipher how pyroM*ϕ*‐EVs shuttled HMGB1 influence TDSCs, we searched for the downstream binding molecules of the EVs‐shuttled HMGB1. By means of the STRING database, we constructed the protein–protein interaction (PPI) network for HMGB1 (**Figure** **7**A). A network type of physical subnetwork with high confidence (minimum required interaction score = 0.7) was selected. Among the top 10 molecules predicted to interact with the HMGB1 physically, TLR9 was suggested to locate at the endosome upon stimulation and thus acquired the opportunity to receive the EVs transported HMGB1, which aroused our interest for investigation. Consistently, through molecular docking, we also validated the direct physical binding ability between HMGB1 and TLR9 in silico (Figure 7B). Then, coimmunoprecipitation was performed for HMGB1 and TLR9 in TDSCs that were treated with different preprocessed EVs. The results verified the physical association between the HMGB1 and TLR9, which was prompted by pyroM*ϕ*‐EVs rather than M*ϕ*‐EVs, and showed reinforcement upon OV‐EVs treatment but showed attenuation upon KD‐EVs treatment (Figure 7C; and Figure S12A,B, Supporting Information). Likewise, the interaction between HMGB1 and TLR9 was weakened upon inhibpyroM*ϕ*‐EVs treatment when compared with the pyroM*ϕ*‐EVs treatment (Figure 7D; and Figure S12C,D, Supporting Information). Using immunofluorescent staining, we next visualized the interaction between the pyroM*ϕ*‐EVs shuttled HMGB1 and TLR9. After pyroM*ϕ*‐EVs treatment, it could be observed that internalized HMGB1 in TDSCs was received by the TLR9 and showed colocalization in the endosome‐like structure (Figure 7E). Further, in the murine burn/tenotomy model, at 7 days after tendon injury, the enhanced expression and colocalization of HMGB1 and TLR9 were also observed when compared with the sham‐operated tendon (Figure 7F,G).

### 2.8 Pyroptotic Macrophage‐Driven Release of IL‐1*β* and HMGB1‐Containing EVs Promoted Senescent and Osteogenic Tendency of TDSCs During Trauma‐Induced HO Formation in an NF‐*κ*B‐Dependent Manner

**Figure 8:**
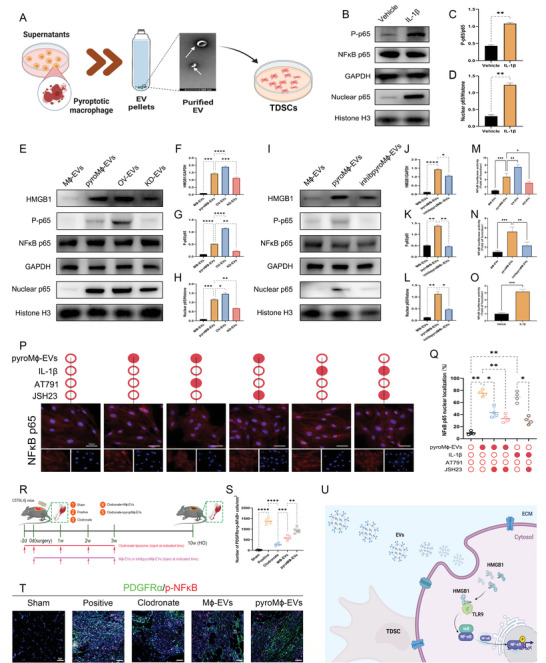
Pathological response of TDSCs to IL‐1*β* and HMGB1‐containing EVs from pyroptotic macrophages converged on NF‐*κ*B signaling. A) Illustration of in vitro experimental protocols. B) WB analysis for expression of HMGB1, NF‐*κ*B p65, and P‐NF‐*κ*B p65 in the total protein or NF‐*κ*B p65 in the nuclear protein of TDSCs upon IL‐1*β* treatment. Quantification of C) relative phosphorylation ratio of P‐NF‐*κ*B p65 to NF‐*κ*B p65 and D) relative protein level of P‐NF‐*κ*B p65 that normalized to Histone H3. E,I) WB analysis for expression of HMGB1, NF‐*κ*B p65, and P‐NF‐*κ*B p65 in the total protein or NF‐*κ*B p65 in the nuclear protein of TDSCs upon indicated EVs treatment. Quantification of F,J) relative protein level of HMGB1 that normalized to GAPDH, (G, K) relative phosphorylation ratio of P‐NF‐*κ*B p65 to NF‐*κ*B p65, H,L) relative protein level of P‐NF‐*κ*B p65 that normalized to Histone H3. M,N,O) Luciferase assay for NF‐*κ*B p65 transcriptional activity determination after indicated treatments. P) Immunofluorescence staining for NF‐*κ*B p65 in TDSCs after indicated treatments and Q) quantification of the NF‐*κ*B p65 nuclear localization fraction. *N* = 3 per group. R) Illustration of the experimental design in the murine burn/tenotomy model. S,T) Analysis and quantification of PDGFR*α*+p‐NF‐*κ*B p65 cells in the regenerative tendon tissues by double immunofluorescence staining at 7 days after indicated treatment. *N* = 6 per group. U) Schematic diagram of exosomal HMGB1 traveled to the TDSCs and provoked TLR9‐meadited NF‐*κ*B p65 signaling. Data are presented as mean±SD. One‐way ANOVA followed by Tukey's test F–H,J–N,Q,S) and unpaired two‐tailed student's *t*‐test C,D,O). *p<0.05, **p<0.01, ***p<0.001, and ****p<0.0001.

Since NF‐*κ*B signaling has been reported as the classical mediator for the Toll‐like receptor signaling, so did it for the IL‐1*β*, we probed whether NF‐*κ*B signaling was linked to the effects exerted by the IL‐1*β* and HMGB1‐containing EVs. Upon addition of pyroM*ϕ*‐EVs or IL‐1*β*, upregulation of NF‐*κ*B signaling emerged in the TDSCs, as manifested by the increased NF‐*κ*B p65 nuclear translocation in WB analysis and immunofluorescent staining, which could be reversed by the TLR9 inhibitor AT791 or the NF‐*κ*B p65 translocation inhibitor JSH23 (**Figure** **8**A,B,D,E,H,I,L). An enhanced phosphorylation ratio of NF‐*κ*B p65 was also found after pyroM*ϕ*‐EVs or IL‐1*β* processing, which is a prerequisite for its transcriptional activity (Figure 8B,C,E,G,I,K). Coherently, the Luciferase assay unraveled that both pyroM*ϕ*‐EVs and IL‐1*β* considerably propelled NF‐*κ*B transcriptional activity, as shown by the boosted luciferase activity driven by the NF‐*κ*B promoter (Figure 8M–O). Such changes in upregulated NF‐*κ*B signaling showed a further intensification in the counterparts that received OV‐EVs, but impairment in the counterparts that received KD‐EVs (Figure 8E,G,H). Similarly, in the group of inhibpyroM*ϕ*‐EVs, the augmentation of NF‐*κ*B signaling was weakened when compared to the counterparts in the pyroM*ϕ*‐EVs group (Figure 8I,K,L), suggesting the promoting and jointed effects of pyroM*ϕ*‐EVs and IL‐1*β* on NF‐*κ*B signaling that function as a downstream effector of HMGB1 and TLR9. In addition, just as expected, administration of the pyroM*ϕ*‐EVs or OV‐EVs led to the elevation of the HGMB1 protein level in the recipient TDSCs, while the increment of HMGB1 was less evident after administration of inhibpyroM*ϕ*‐EVs. Nonetheless, the HGMB1 mRNA levels kept consistent among EVs treated groups (Figure 8E,F,I,J; and Figure S10H,I, Supporting Information). Thus, we could infer that pyroM*ϕ*‐EVs could shuttle the HGMB1 to modulate the signaling and function of TDSCs rather than influence the transcription of HGMB1.

Coinciding with the in vitro findings, NF‐*κ*B signaling was also conspicuously activated in osteogenic progenitors during the trauma‐induced HO formation in the murine model, which was demonstrated by the increased PDGFR*α*+P‐NF‐*κ*B+ cells density after injury through double immunofluorescence staining. Clodronate liposome administration significantly diminished the density of PDGFR*α*+P‐NF‐*κ*B+ cells, which was mimicked by the IL‐1*β*‐neutralizing antibody addition but was rescued by the pyroM*ϕ*‐EVs supplementation, providing the in vivo evidence that pyroptotic macrophages‐derived EVs or IL‐1*β* could heighten NF‐*κ*B activation in TDSCs (Figure 8R–T; and Figure S13A–C, Supporting Information). The trafficking and action mechanism of EVs‐shuttled HMGB1 is summarized (Figure 8U).

**Figure 9:**
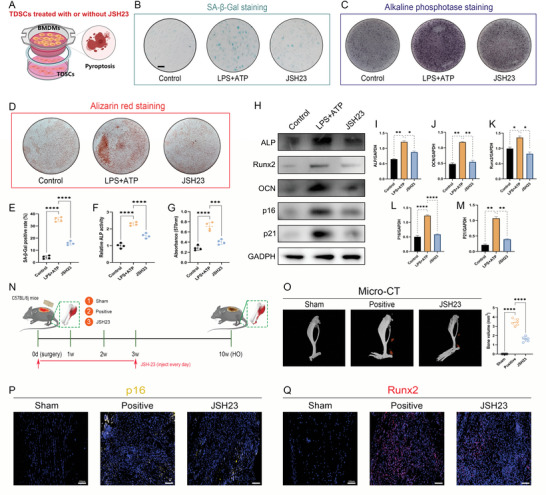
NF‐*κ*B signaling was responsible for the pathogenic effects of pyroptotic macrophages on TDSCs during trauma‐induced HO formation. A) Illustration of the in vitro coculture system and experimental treatment design. B) SA‐*β*‐Gal staining for TDSCs after indicated treatments. C) ALP staining for TDSCs following indicated treatments at 7 days after osteogenic induction. D) ARS staining for TDSCs following indicated treatments at 21 days after osteogenic induction. E) Quantification of SA‐*β*‐Gal positive rate. F) Quantification of ALP activity for TDSCs following indicated treatments at 7 days after osteogenic induction. G) Quantification of absorbance of eluted ARS staining at 570 nm. *N* = 4 per group. H) WB analysis for cellular senescence marker p16, p21, and osteogenic marker Runx2, ALP, and OCN. I–M) Quantification of protein level of p16, p21, Runx2, ALP, and OCN normalized to GAPDH. *N* = 3 per group. N) Illustration of the experimental design in the murine burn/tenotomy model. O,P) Micro‐CT analysis and quantification for HO formation following indicated treatments at 10 weeks after injury. Q) Immunofluorescent staining of p16 in regenerative tendon tissues following indicated treatments at 7 days after injury. R) Immunofluorescent staining of Runx2 in regenerative tendon tissues following indicated treatments at 7 days after injury. *N* = 6 per group. Data are presented as mean±SD. One‐way ANOVA followed by Tukey's test. *p<0.05, **p<0.01, ***p<0.001, and ****p<0.0001.

JSH‐23 was then used for further confirmation of the contribution of NF‐*κ*B signaling. As anticipated, the addition of the JSH‐23 rejuvenated the senescent TDSCs induced through coculturation with the pyroptotic macrophages, unfolded as the reduction in the proportion of the SA‐*β*‐Gal+ cells and senescent cellular marker (**Figure** **9**A,B,E,H,L,M). Simultaneously, JSH‐23 decelerated the osteogenic induction of the TDSCs in the coculture model that was measured by the ALP, ARS staining combined with the WB analysis for osteogenic indicators (Figure 9C,D,F–M). For the application of JSH‐23 in the murine trauma‐induced HO model, inhibition of the NF‐*κ*B signaling also effectively diminished the senescent cells burden in the injury site and abrogated the eventual HO formation (Figure 9N–Q). Therefore, these results confirmed that NF‐*κ*B signaling was involved in the trauma‐induced HO course that was actuated by the pyroptotic macrophages.

Finally, the activation of the HMGB1/TLR9/NF‐*κ*B axis was examined in the clinical samples to establish the clinical relevance of the findings. Soft tissue‐derived HO samples and control soft tissue samples (normal tendon) were collected from respective age, sex, and BMI‐matched cohort. HE and Masson's trichrome staining exhibited their typical histological features, through which the regenerated ligament region and ossification region were distinguished and defined on HO samples (Figure S14A,B, Supporting Information). As a result, Stronger immunohistochemical staining for HMGB1, TLR9, and phosphorylated NF‐*κ*B p65 (p‐NF‐*κ*B) were found in the HO tissues than those in the normal tendon tissues, just like the expression pattern in the mice. Noteworthily, even within the HO tissues, expression of HMGB1, TLR9, and p‐NF‐*κ*B in the ossification region was greater than those in the ligament region, while the overall staining intensity of these molecules in the ligament region was still at a higher level when compared with those in the normal tendon region (Figure S14C, Supporting Information). Taken together, these data suggested that cells in the human HO tissues were in an activated state of HMGB1/TLR9/NF‐*κ*B signaling for ossification, showing a similar trend with the murine tissue section.

## 3 Discussion

**Figure 10:**
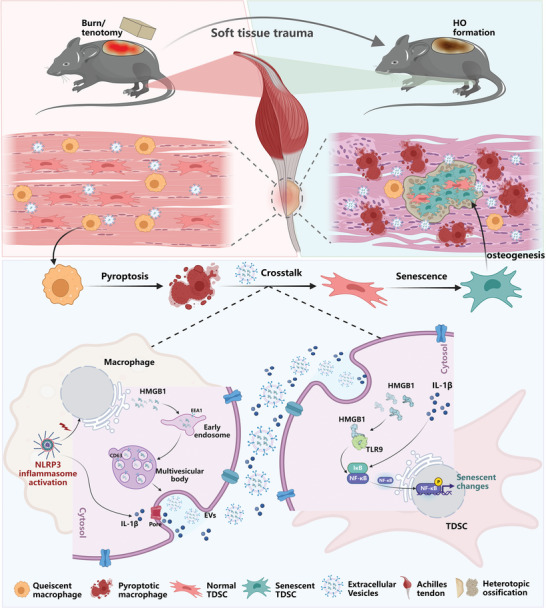
Schematic depiction of the crosstalk between pyroptotic macrophage and senescent TDSCs to prompt aberrant osteogenic healing during trauma‐induced heterotopic ossification.

HO which occurs in the form of unfavored osteochondral formation within soft tissues, is actually a frustrating healing result in response to severe trauma, which severely hampers the structure and function of the extremity.^[^ ^21^, ^22^ ^]^ Such an erroneous repair, in fact, tends to be the reasonable result of the defective coordination of the regenerative components upon soft tissue injury. Given that stem/progenitor cells account for the main regenerative forces through theirs self‐renewal and lineage commitment capacity, it is assumed that the defective regenerative mechanism might be largely ascribed to the incompetence of mesenchymal stem cells (MSCs), which, however, could be firmly dictated by the residing niche.^[^ ^23^, ^24^ ^]^ Multiple studies have already suggested that immune system activity was dysregulated during the HO progression, contributing to an osteogenesis‐permissive niche formation.^[^ ^25^ ^]^ In our previous study, we also proved that macrophages played a potent role in tissue‐remodeling cytokines releasing and initiating the HO formation, though the downstream mechanism still warrants further investigation.^[^ ^14^ ^]^ In this study, we demonstrated that macrophage pyroptosis appeared in accompany by the senescence of TDSCs after the tendon injury, presenting an unprecedented crosstalk paradigm between macrophages and TDSCs where the pyroptotic macrophages induced the senescence of TDSCs and thus contributed to the HO formation. Both IL‐1*β* and HMGB1 containing EVs were confirmed to be involved in the contributing factors of pyroptotic macrophages to the TDSCs aberrant differentiation, with NF‐*κ*B signaling identified as the underlying mechanism (**Figure** **10**). Since there is a lacking of effective therapeutics for HO treatment, our findings in this study may reveal novel targets and pave the footholds for future treatment strategies designing.

Aging has been commonly recognized as a predisposing factor to the defective regenerative capacity of the elderly population.^[^ ^26^, ^27^ ^]^ Cellular senescence appears during aging, severely disrupts tissue homeostasis, and alters the regenerated tissue profile, as shown in plenty of age‐related disorders like osteoarthritis and atherosclerosis. Notably, in recent times, studies revealed that cellular senescence could also take place in acute injury scenarios. Increased senescent cell burden could be found after skeletal muscle injury and traumatic brain injury.^[^ ^28^, ^29^ ^]^ In the present study, we similarly found that senescent cells accumulated in response to the tendon injury. Further, we figured out the identity of these senescent cells involved in osteoprogenitors. Particularly, the senescence of progenitor cells could arguably make a good explanation for the erroneous repair machinery of the soft tissues during HO formation. As manifested in osteoarthritis, senescent stem cells lost their chondrogenic potential, while in osteoporosis, senescent bone marrow stem cells shifted lineage commitment propensity and preferentially differentiated into adipocytes instead of osteoblasts.^[^ ^30^, ^31^ ^]^ Alternatively, the elimination of senescent cells from oxidatively stressed TDSCs largely relieved their osteogenic potential as quoted from this study. When it comes to tendon biology, it was also demonstrated that senescent TDSCs showed altered lineage commitment, favoring osteogenic over tenogenic.^[^ ^32^, ^33^ ^]^ Therefore, the wrong fate decision of senescent TDSCs might be responsible for the HO‐directed aberrant osteochondral healing. Additionally, SASP might comprise another machinery linking senescent cells to tissue reprograming after soft tissue injury. A study conducted by Monteiro et al. established that senescent cells could produce IL‐6 to activate the regenerative cues for tissue reprogramming.^[^ ^34^ ^]^ Interestingly, this machinery has also been validated in the murine FOP model by wang et al.^[^ ^12^ ^]^ They cocultured myoblasts with senescent cells and found that senescent cells supported the chondrogenic differentiation of myoblasts of the FOP origin through contributing soluble factors. Hence, we could conclude from the above evidence that the accumulating senescent TDSCs after the tendon injury served a vital role in the aberrant osteochondral healing process during HO formation, either intrinsically or extrinsically. In the following experiments, we next aimed to find out the contributing factors to the TDSC senescence during HO formation.

Inflammation has been widely shown to be critical in inducing premature senescence in traumatic diseases. Essentially, even in the natural aging process, it is supposed that the chronic low‐grade inflammation raised by the hypersensitive aged host defense system (termed inflamm‐aging) contributed to the extensive cellular senescence.^[^ ^35^, ^36^ ^]^ Macrophages are potent inflammation makers from innated immunity and meanwhile master players in both soft tissue development and regeneration.^[^ ^37^, ^38^ ^]^ Despite well‐described dysregulated macrophage activity in trauma‐induced HO, little is known regarding how it worked to facilitate aberrant healing versus normal regeneration.^[^ ^39^ ^]^ Inflammasome‐mediated pyroptosis has recently been realized as a frequent way for macrophages to trigger inflammation, which prompts us to study its role in trauma‐induced HO. In the present study, we disclosed that macrophage pyroptosis is induced after tendon injury, which is anatomically accompanied by increased senescent cell burden. Then, genetic ablation of pyroptosis was performed using NLRP3−/− mice to verify its role in HO formation. The results exhibited that senescent cell burden and HO formation were obviously mitigated in NLRP3−/− mice, indicating pyroptosis as a vital event for HO development. In view of the diversity of cell types and their complex cellular interplay involved in the injury niche, to establish a more direct causal relationship between macrophage pyroptosis and TDSC senescence, we cocultured TDSCs with pyroptotic macrophages in vitro. As expected, coculture with pyroptotic macrophages potently promoted senescence and osteogenic induction of TDSCs, which was largely impaired when NLRP3−/− macrophages were applied. Thus, we could suggest that cellular senescence might be educated by the pyroptotic macrophages and mediating the effects of macrophages on tissue reprogramming. Regrettably, due to the focus on the stem cell biology dictated by the pyroptotic macrophages, we did not specifically investigate the effect of TDSCs on macrophages in this system. However, from literature, we could find that SASP released by senescent cells was capable of educating the non‐committed macrophages to the pro‐inflammatory subtype resembling the classical M1 polarization.^[^ ^40^ ^]^ Besides, a previous report also suggested that senescent cells could disable the efferocytosis function of macrophages to enable their coexistence within the tissue milieu.^[^ ^41^ ^]^ Therefore, we could propose that crosstalk between senescent cells and pyroptotic macrophages might result in positive feedback and thereby led to delayed and persistent inflammation in the injured wound, which caused an aberrant healing response.

Proinflammatory cytokines make up a vital part of the soluble fractions that are indispensable for the paracrine function of macrophages. Among these soluble cytokine mediators, IL‐1*β* was the featured pleiotropic pro‐inflammatory cytokine that was produced upon the macrophage pyroptosis.^[^ ^42^ ^]^ Actually, IL‐1*β* was already reported to be effective in inducing osteogenic and degenerative changes in musculoskeletal disorders.^[^ ^43^, ^44^ ^]^ In the preceding study with respect to HO, IL‐1*β* was also proposed to be the powerful culprit for inducing aberrant osteogenic behavior of TDSCs, which conformed to the results of our study, where the rescue experiments using an antibody against IL‐1*β* reinforced this argument.^[^ ^21^ ^]^ Further, this study revealed that IL‐1*β* was a key mediator of the pro‐senescent effect of pyroptotic macrophages on TDSCs during HO formation, and could take effect alone as an initiator or in conjunction with other proinflammatory factors as a reinforcer However, unlike TNF‐*α*, which could be conventionally secreted through the endoplasmic reticulum‐Golgi secretory pathway, the significant exit of IL‐1*β* required inflammasome assembly to both cleave it to the active form and generated cell membrane pores to facilitate its escape. Thus, this study additionally added the rationale behind IL‐1*β* release during HO formation since pyroptosis induction triggered inflammasome assembly. Surprisingly, IL‐1*β* neutralizing did not cause a dramatic, though significant, inhibitory effect on HO formation in the murine models, which may argue against its significant role in the HO. However, Sorkin et al. suggested that TGF*β* depletion in macrophages can be compensated by the feedback response, which minimized the effects of TGF*β* depletion and provided a hint to the case of IL‐1*β* neutralizing.^[^ ^25^ ^]^ It might be plausible that IL‐1*β* neutralizing also aroused other proinflammatory reactions that confused the intervention outcome. Thus, the importance of IL‐1*β* may be underestimated by the present results in this study.

EVs are the nanosized membrane‐bound vehicles (30–150 nm in diameter) typically released by cells as an important intercellular communicator.^[^ ^45^ ^]^ Considering the pivotal action of EVs in transferring regulatory signals within the microenvironment, their role in mediating the effects of pyroptotic macrophages during HO should not be ignored. In this study, we also concluded that EVs from pyroptotic macrophages are crucial for senescent TDSCs induction, which was demonstrated by the impaired TDSCs senescence by pyroptotic macrophages after pharmacological blocking of EVs secretion. In contrast, the direct addition of pyroptotic macrophage‐derived EVs aggravated the senescent TDSCs load. In addition to that, it followed with the question that what effector contents enclosed in the EVs are mediating this pro‐senescent effect. As suggested by the literature, various DAMP molecules could be abundantly generated upon pyroptosis.^[^ ^20^, ^46^ ^]^ HMGB1 is one of these DAMP molecules that has been documented to be closely associated with cellular senescence.^[^ ^47^, ^48^ ^]^ At steady state, HMGB1 resides in the nucleus to act as a DNA chaperone for maintaining nucleus homeostasis but is reported to exit from the cell nucleus and be released to the extracellular space as a result of the programmed death pathways, including that in the context of pyroptosis.^[^ ^49^ ^]^ Of note, one proven route of HMGB1 release from activated macrophages is through the vesicles.^[^ ^50^ ^]^ On these grounds, we tried to figure out whether HMGB1 was involved in the cargo of pyroM*ϕ*‐EVs and thus served as an intermediary for TDSCs senescence induction. Our results proved that HMGB1 was enriched in the EVs isolated from pyroptotic macrophages. Consistent with this, HMGB1 expression was significantly enhanced in the murine traumatic HO model, which localized with the EVs marker CD63 and macrophages marker F4/80, indicating that HMGB1 could contribute to the HO formation through the macrophage‐derived vesicles in vivo. Additionally, inspired by the predicted PPI network generated with the STRING algorithm, together with the cellular location of these molecules, we secured TLR9 as our target receptor of interest to HMGB1 for investigation. Through molecular interaction analysis both in vitro and in vivo, we validated the key role of TLR9 in transmitting the function of HMGB1‐containing pyroM*ϕ*‐EVs, and highlighted the significance of TLR in mediating Trauma‐induced HO, a common feature also shared by other inflammation‐related diseases.^[^ ^51^, ^52^ ^]^

Concerning the succeeding molecular mechanism, it has been suggested in diabetic retinopathy and inflammatory bowel disease that HMGB1 activated the NF‐*κ*B pathway, which also tends to be the crucial executor of the TLR‐based signaling.^[^ ^53^, ^54^ ^]^ In keeping with our previous study, we again verified elevated NF‐*κ*B signaling as the vital molecular driver for trauma‐induced HO formation, but this time as the downstream event of the HMGB1‐TLR9 axis.^[^ ^14^ ^]^ Coincidently, IL‐1*β* also led to NF‐*κ*B signaling activation in TDSCs, thus converging with the pyroM*ϕ*‐EVs to induce cellular senescence and aberrant osteogenic differentiation of TDSCs. In line with these findings, prior studies also determined NF‐*κ*B is signaling to be imperative for the cellular senescence within the degenerative rotator cuff and HO formation within the rat traumatic HO model.^[^ ^55^, ^56^ ^]^

Certain limitations are involved in this study. First, Though the significance of cellular senescence in HO formation after soft tissue injury was validated in this study, how senescent cells drive the aberrant osteochondral healing and HO formation need to be carefully probed in the future study; Second, HMGB1 might not be the sole effect‐mediating molecule of the pyroM*ϕ*‐EVs, thus other components of EVs that should be further searched with the help of some high‐throughput methods through HMGB1 was also screened and ranked top in the previously reported proteomic results.^[^ ^20^ ^]^ By the same token, other receptors for HMGB1 except TLR9 should be testified. Besides, the complex forming of HMGB1 and TLR9 is often assisted by the DNA. Whether pyroM*ϕ*‐EVs also incorporated DNA was worthwhile to determine since there was evidence that EVs could deliver parent cell‐derived‐genomic DNA.^[^ ^57^ ^]^ Third, despite the application of the general knockout mice model in the present study, the cell type selectivity is absent, and there remains the possibility that pyroptosis silencing in other cells may confuse the results. Hence, future research using a conditional knockout murine model is encouraged. Fourth, in addition to the crosstalk between the inflammatory macrophages and stem cells, the communication of other inflammatory cells and even the systemic inflammation to the stem cell biology are worthy of deeper investigation.

In summary, our study found that macrophage pyroptosis was severely induced after tendon injury, which facilitated the TDSCs senescence and aberrant osteochondral healing to cause HO formation. Mechanistically, pyroptotic macrophages secreted IL‐1*β* and HMGB1 containing EVs, synergistically instigating the senescence of TDSCs during HO progression. Our findings thus provide ponderable insights into HO therapeutic strategy.

## 4 Experimental Section

### Primary Culture of BMDMs

To isolate BMDMs, C57BL/6 male mice aged 7–8 weeks were sacrificed to harvest bone marrow as described before.^[^ ^58^ ^]^ Briefly, the femur and tibiae were excised from mice, and the *α*‐minimum essential medium was used to flush the bone marrow out to the culture plates. After removing the erythrocytes with erythrocytes lysis buffer (Yeasen, China), the bone marrows were reconstituted in complete RPMI 1640 medium supplemented with 10% fetal bovine serum (FBS) (Biological Industries, Beit Haemek, Israel), 1% w/v penicillin/streptomycin and 40 ng mL^−1^ M‐CSF (Peprotech, NJ) and cultured for 3 days. Nonadherent cells were then removed, and the culture medium was refreshed to incubate for additional 3 days in the presence of 40 ng mL^−1^ M‐CSF. After reaching 80% confluence, the cells were harvested by scraping for further use. For subsequent experiments, a medium containing the 20 ng mL^−1^ M‐CSF was applied for maintaining BMDMs survival.

For pyroptosis induction, BMDMs were treated with 1 µg mL^−1^ lipopolysaccharide (LPS) for 3 h and then 5 mm ATP for 1 h. For IL‐1*β* neutralization, BMDMs were treated with 1 µg mL^−1^ lipopolysaccharide (LPS) for 3 h, followed by 5 mm ATP for 1 h in the presence of 150 ng mL^−1^ IL‐1*β*‐neutralizing antibody. For EVs secretion blocking, BMDMs were pretreated with 10 µm GW4869 for 24 h and then stimulated with 1 µg mL^−1^ lipopolysaccharide (LPS) for 3 h, followed by 5 mm ATP for 1 h.

### Isolation and Culture of TDSCs

The isolation of TDSCs was performed according to a previous report with minor modifications. Briefly, C57BL/6 male mice aged 2–3 weeks were selected, and their tail tendon tissues were excised. The tail tendon was first cut into multiple small pieces and then subjected to digestion in the presence of 3 mg mL^−1^ type I collagenase (Worthington) and 4 mg mL^−1^ type II dispase (Worthington) for 2 h at 37 °C. The digestion solution was filtered through a 70 µm strainer and concentrated at 1000 rpm for 5 min for cell pellets collection. After being washed with phosphate buffer solution (PBS), the collected cells were again precipitated and resuspended in the complete *α*‐MEM containing 10% FBS. The cells were then cultured in the carbon dioxide incubator at 37 °C with the culture medium changed every 2 days. For the downstream studies, only TDSCs within the 3 passages were used.

For signaling pathway regulation, TLR9 blocking was performed by pretreatment TDSCs with AT791 (1 µm) for 4 h and subjected to indicated treatments. Inhibition of NF‐*κ*B p65 nuclear translocation was performed by pretreatment TDSCs with JSH23 (10 µm) for 1 h and subjected to indicated treatments

### Cell Transfection

For HMGB1 overexpression, the lentivirus vector encoding murine HMGB1 was synthesized by Genomeditech (Shanghai, China). Transfection was conducted in line with the provider's instruction. The multiplicity of infection (MOI) of 20:1 was applied for transfection in combination with 5 µg mL^−1^ polybrene. After 72 h of transfection, cells were used for downstream experiments.

For HMGB1 knockdown, siRNA targeting murine HMGB1 was used. The sequences of three candidate HMGB1 siRNA were listed in Table S1 (Supporting Information). Lipofectamine RNAiMAX Reagent (Invitrogen, USA) was employed for BMDM transfection as per the manufacturer's instruction. The efficiency of knockdown was evaluated, and experiments were conducted after 48 h of transfection.

### Coculture Assay

For the coculture assay, the transwell system (Corning, USA) was adopted with a porous membrane pore size selected to be 0.4 µm. BMDMs were inoculated at the upper chamber of the transwell system, while TDSCs were cultured at the bottom chamber at a ratio of 4:1.

### Osteogenic Differentiation of TDSCs

The isolated TDSCs were plated onto the 6‐well plates and maintained growth until the confluence reached 80–90%. Then the culture medium was changed into the osteogenic differentiation medium (Cyagen, China) for osteogenic induction.

After osteogenic induction for 7 days, ALP staining was performed with BCIP/NBT Alkaline Phosphatase Color Development Kit (Beyotime, China) as per the manufacturer's instructions. ALP activity was also determined using the alkaline phosphatase assay kit (Nanjing Jiancheng Biotechnology Co Ltd, China). After 21 days of osteogenic induction, alizarin red S (ARS) staining was performed with a 2% Alizarin Red S (pH 4.2) solution. For the ARS staining quantification, 10% cetylpyridine chloride was used to dissolve the ARS dye and transferred it to the 96‐well plates. Absorbance at 570 nm was recorded by SpectraMax i3x (Molecular Devices, Australia).

### Senescence Associated *β*‐Galactosidase (SA‐*β*‐Gal) Staining

SA‐*β*‐Gal staining kit was applied for the SA‐*β*‐Gal staining in accordance with the manufacturer's instructions. Briefly, cells were fixed with 4% paraformaldehyde for 15 min at ambient temperature. The SA‐*β*‐Gal staining solution was prepared following the manual instruction and incubated with fixed cells for 16 h at 37 °C. Images of six random fields in each well were captured and the percentage of the SA‐*β*‐Gal positive (SA‐*β*‐Gal+) cells was calculated and referred to as the SA‐*β*‐Gal positive rate.

### Cellular Immunofluorescence Staining

After plating onto a 24‐well plate and treating with different regimens, cells were fixed in 4% paraformaldehyde for 20 min prior to permeabilization with 0.5% Triton X‐100 for 15 min. 1% BSA was used for blockade for 1 h at room temperature. Then, corresponding primary antibodies were used to incubate the cells at 4 °C overnight. The next day, cells were washed three times with phosphate‐buffered saline with Tween20 (PBST), and secondary antibodies were added for 1 h at room temperature in the dark, DAPI (4ʹ,6‐diamidino‐2‐phenylindole; Beyotime, China) staining was performed to counterstain the cell nucleus. Fluorescence was observed using Olympus DP70 inverted microscope (Japan), and representative images were obtained. The primary antibodies used in this study are listed in Table S2 (Supporting Information).

### EVs Isolation and Characterization

EVs‐depleted serum was acquired by ultracentrifugation of the FBS (Gibco, USA) for 18 h at 100 000 g, and the upper four‐fifths of the supernatant was retained. For EVs isolation, BMDMs were washed two times with PBS before incubation in cultured in RPMI 1640 medium supplemented with 10% EVs‐depleted serum for 48 h. Then, supernatants from unstimulated macrophages and pyroptotic macrophages were harvested and centrifuged at 300 g for 10 min, 2000 g for 20 min, and 10 000 g for 30 min at 4 °C for cells and cell debris removal. Subsequently, supernatants were collected and filtered through 0.22 µm filters before further ultracentrifuging at 120 000 g for 70 min at 4 °C and repeated for two rounds. Pelleted EVs were resuspended in PBS and stored at −80 °C until usage. The EVs derived from unstimulated macrophages and pyroptotic macrophages were referred to as M*ϕ*‐EVs and pyroM*ϕ*‐EVs, respectively.

The morphology of the EVs was observed by TEM. Particle size distribution and concentration were characterized through NTA (ZetaView PMX 120, Particle Metrix, Meerbusch, Germany). WB analysis of CD9, CD63, TSG101, ALIX, F4/80, and calnexin was also applied for validation of the successful isolation of EVs.

### Dil Labeling of EVs

For visualization of the EVs internalization, labeling of EVs with Dil dye was performed. Briefly, isolated EVs were incubated with 2 µm Dil dye staining working solution (Molecular Probes, USA) for 10 min. Then, the excess Dil dye was removed by ultracentrifuging at 12 000 g for 70 min. The obtained labeled EVs were then washed in PBS, ultracentrifuged at 12 000 g for 70 min for collection, and further filtered through a 0.22 µm membrane strainer.

EVs internalization assay was performed by incubating TDSCs with Dil‐labeled EVs for 24 h. iFluor 488‐conjugated phalloidin (Abcam, USA) was used for staining the cytoskeleton. DAPI was used for counterstaining. Images were captured by a fluorescence microscope (Eclipse TS100; Nikon Corporation, Tokyo, Japan).

### Molecular Modeling and Docking Analysis

The structures of murine Toll‐like receptor 9 (TLR9) (4QDH) originated from RCSB Protein Data Bank, and HMGB1 (AF_AFP63158F1) was acquired from the AlphaFold Protein Structure Database. Then, the Hdock software (http://hdock.phys.hust.edu.cn/) was applied for docking experiments and generating the predicted protein binding mode. The docking results were finally visualized in the PyMOL software and shown.

### Human Samples Collection

The Institutional review board of Shanghai Sixth People's Hospital Affiliated to Shanghai Jiao Tong University School of Medicine approved the study (2022‐KY‐004). Written informed consent was acquired and signed by the patients or their immediate family members. Healthy individuals aged 19–55 were enrolled in this study who suffered from HO formation following their prior internal fixation surgeries for elbow fractures. No local radiotherapy was given to the patients. The HO samples were collected during their combined elbow arthrolysis surgery. All harvested HO samples in this study are at their maturation stage in light of the ethical consideration that surgery was only performed when the HO became mature. The normal tendon served as the control tissue for HO since HO originates from the soft tissues, including the tendon, which was obtained from the age‐matched healthy individuals who received the anterior cruciate ligament reconstruction surgery. The excess normal hamstring tendon after the usage for anterior cruciate ligament reconstruction was harvested. The demographic information of the involved patients from the two groups was summarized, and no significant differences were detected in any confounding factors (gender and BMI) that have been previously identified to impact the occurrence rate of traumatic HO.^[^ ^59^ ^]^

### Animals and Models

Wild‐type C57BL/6 mice were provided by the Shanghai laboratory animal center (SLAC), while the NLRP3 general knock‐out (NLRP3−/−) mice were acquired from the Jackson laboratory (stock No. 02 1302) and housed in the specific pathogen‐free facility. Primers used for genotyping the NLRP3−/− mice were listed in Table S3 (Supporting Information).

The murine burn/tenotomy model was generated as the trauma‐induced heterotopic ossification model according to previously reported methods.^[^ ^60^, ^61^ ^]^ Only male mice were employed for modeling since there might be differential immune responses between male and female mice and thus create data variability. Briefly, male C57BL/6 mice aged 7–8 weeks were anesthetized with 1% pentobarbital sodium. The medial aspect of the distal hindlimb was chosen to make a 0.5 cm longitudinal cut for the exposure of the Achilles tendon. Then a complete transverse cut was made on the midpoint of the Achilles tendon without stitching. Skin closure was performed with 5‐0 Vicryl stitches. After the abovementioned tenotomy, the Dorsal skin of all mice was carefully shaved to leave ≈30% of the body surface naked areas. An aluminum block (2 × 2 × 3 cm^3^, weighing 35 g) was heated to 60 °C via a water bath and then placed onto the naked dorsal skin of mice for 17 s to create the burn injury. For the Sham surgery, the incision was only made on the skin of the distal hindlimb, with the Achilles tendon remaining intact. All animals were subjected to surgeries after at least 1 week of accommodation to the environment. Free access to food and water is provided. To minimize cage‐to‐cage variation, the number of mice placed in each cage was adjusted to be close after every sacrifice.

For the assessment of the role of pyroptosis in the formation of HO, both wild‐type and NLRP3−/− mice were subjected to burn/tenotomy modeling (*n* = 12). Wild‐type mice that only received sham surgery served as control (*n* = 12).

For the assessment of the role of the macrophage‐derived EVs in the formation of HO, mice were randomly assigned into four groups (*n* = 12): Sham group (received Sham surgery), Positive group (received burn/tenotomy and PBS), Clodronate group (received burn/tenotomy surgery and clodronate liposome), M*ϕ*‐EVs group (received burn/tenotomy surgery, clodronate liposome, and M*ϕ*‐EVs), pyroM*ϕ*‐EVs group (received burn/tenotomy surgery, clodronate liposome, and pyroM*ϕ*‐EVs), and inhibpyroM*ϕ*‐EVs group (received burn/tenotomy surgery, clodronate liposome, and inhibpyroM*ϕ*‐EVs). Mice in the Clodronate group were first injected with clodronate liposome (1.33 mL 100 g^−1^) intraperitoneally 2 days before surgery, then, clodronate liposome (100 µL) was locally delivered into the tenotomy site at the time of surgery. Weekly intraperitoneal injection (1.33 mL 100 g^−1^) was thereafter performed for 2 weeks. Mice in the M*ϕ*‐EVs group, pyroM*ϕ*‐Evs group, or inhibpyroM*ϕ*‐EVs group were locally administrated with M*ϕ*‐EVs, pyroM*ϕ*‐EVs, or inhibpyroM*ϕ*‐EVs (20 µg in 10 µL PBS) respectively in addition to peritoneally administration of clodronate liposome weekly until 3 weeks, whereas mice in the Sham and Positive group were administrated with PBS using the same strategy as the M*ϕ*‐EVs group and pyroM*ϕ*‐EVs group.

For the assessment of the role of HMGB1 in the macrophage‐derived EVs, mice were also randomly assigned into five groups (*n* = 12): Sham group (received Sham surgery), Positive group (received burn/tenotomy and PBS), GW4869 group (received burn/tenotomy surgery and GW4869), HMGB1‐OV‐EVs group (received burn/tenotomy surgery, GW4869, and HMGB1‐OV‐EVs) and HMGB1‐KD‐EVs group (received burn/tenotomy surgery, GW4869, and HMGB1‐KD‐EVs). Mice in the Sham and Positive groups received the treatment protocol as described above. Mice in the GW4869 group received the intraperitoneal administration of GW4869 at a dosage of 2.5 mg kg^−1^ d^−1^ every day for 3 weeks. Mice in the HMGB1‐OV‐EVs group or HMGB1‐KD‐EVs group were locally administrated with HMGB1‐OV‐EVs or HMGB1‐KD‐EVs (20 µg in 10 µL PBS) respectively, in addition to peritoneally administration of clodronate liposome weekly until 3 weeks.

For the rescue studies, mice were randomly assigned into the following groups (*n* = 12): Sham group (received Sham surgery), Positive group (received burn/tenotomy and vehicle or isotype control IgG), *α*IL‐1*β* group (received burn/tenotomy and IL‐1*β*‐neutralizing antibody), JSH‐23 group (received burn/tenotomy and JSH‐23). IL‐1*β*‐neutralizing antibody was subcutaneously administrated to the mice at a dosage of 10 µg twice a week for 3 weeks. JSH‐23 was intraperitoneally administrated to the mice at a dosage of 3 mg kg^−1^ d^−1^ every day for 3 weeks. The details of the reagents and chemicals used in this study are listed in Table S4 (Supporting Information).

All the above animal experiments were reviewed and approved by the Institutional Animal Care and Use Committee (IACUC) of Shanghai JiaoTong University Affiliated Sixth People's Hospital (DWLL‐2023‐0373). All experiment reports were performed in compliance with the ARRIVE guidelines (https://arriveguidelines.org/). Investigators were blinded to the group allocation when performing the data analysis.

### Histological, Immunohistochemical, and Immunofluorescent Staining

Tissues for examination were harvested at 7 days and 10 weeks postinjury. Soft tissues from the tendomuscular junction to the enthesis were excised and fixed in 10% formalin. Tissues collected at 10 weeks were further subjected to decalcification in 10% ethylenediaminetetraacetic (EDTA) for 14 days. After paraffin embedding, 5 µm thickness tissue sections from the coronal aspect were obtained and pasted onto the glass slide. Hematoxylin and eosin (HE) staining was performed through a routine process according to the manufacturer's instructions. For immunohistochemical staining, sections were deparaffinized and rehydrated in a series of alcohol gradients. After antigen retrieval in sodium citrate, sections were incubated with 3% hydrogen peroxide for 15 min to deactivate endogenous peroxidase. The sections were then blocked with 10% serum for 1 h and incubated with corresponding antibodies at 4 °C overnight. Then sections were washed three times with PBST before being incubated with biotinylated second antibodies for 1 h at room temperature. SAB complex and diaminobenzidine were added at last for color development. All sections were observed in an Olympus microscope.

For immunofluorescence staining, rehydration, antigen retrieval, and blocking were performed as described in immunohistochemical staining. Sections were incubated with corresponding antibodies at 4 °C overnight before being treated with Alexa Fluor 488‐ or cy3‐conjugated secondary antibody for 1 h at 37 °C. DAPI was applied for counterstaining. Pseudocolor processing was performed for better visualization. For triple immunofluorescence staining, tyramide signal amplification (TSA) was performed. Briefly, after deparaffinized and rehydration, antigen retrieval was performed, followed by blocking. Corresponding primary antibodies were sequentially added before incubation with the horseradish peroxidase (HRP)‐conjugated polymer. Visualization of each antibody was realized using CY3‐TSA, FITC‐TSA, or CY5‐TSA. Antibodies stripping was performed between each antibody detection procedure. At last, nuclei were counterstained with DAPI, and the stained slides were scanned using the Pannoramic digital scanner (Pannoramic P250; 3DHISTECH) or TissueFAXS Spectra Platform. The primary antibodies used in this study are listed in Table S2 (Supporting Information).

### Micro‐CT Scanning

10 weeks after surgery, mice were sacrificed, and hindlimbs were harvested. 10% v/v formalin was applied for hindlimbs fixation for 48 h before the samples were detected by the high‐resolution Micro‐CT scanner Skyscan 1176 (software = Version 1.1 (build 6), Bruker, Kontich, Belgium). Isometric resolution at 18 mm and voltage at 70 kV was selected as the parameter for scanning. CTvox software (Version 3.0.0 r1114) was used for the reconstruction of 3D images of bone anatomy. Bone volume was then analyzed and calculated using CTan software (Version 1.15.4.0+, Bruker) as previously reported (34). Any masses present within the soft tissues with a density above 272, shown on the scanning slices, were determined to be heterotopic bone.

### RNA Sequencing

Total RNA of regenerated tendon tissues (Soft tissues from the tendomuscular junction to the enthesis) and normal Achilles tendon tissues (served as a control) was extracted from the cartilage tissues using Trizol (Invitrogen, CA) following the manual instructions. Briefly, ribosomal RNA (rRNA) was first depleted from the total RNA, followed by RNA fragmentation. The first strand cDNA was subsequently generated through reverse transcription of the cleaved RNA fragments. The RNA template was then removed, and a replacement strand was synthesized during the second strand cDNA synthesis. After the addition of a single “A” base and ligation of the adapter to the aforementioned cDNA fragments, PCR was employed for the final cDNA library creation. Pair‐end sequencing was performed on the Illumina sequencing platform (HiSeqTM2500; Shanghai Oebiotech Co., Ltd., Shanghai, China). Gene expression levels were computed via the reads per kilobase of transcript per million mapped reads method. Differentially expressed genes (DEGs) were identified and filtered with the *p* value < 0.05 and the absolute value of log2 fold change > 1. The heatmap for gene expression was plotted using an online platform (https://www.omicstudio.cn/tool/4). Gene set enrichment analysis (GSEA) was conducted with the GSEA 4.2.2 software (Broad Institute, Cambridge, USA).

### Real‐Time Quantitative Polymerase Chain Reaction (RT‐qPCR)

Total RNA was extracted using either the Tissue RNA Purification Kit PLUS (EZB‐RN001‐plus, EZBioscience, China) or the EZ‐press RNA Purification Kit (B0004DP, EZBioscience) according to the manufacturer's instructions. Following reverse transcription was conducted using the Color Reverse Transcription Kit RNA purification kit (A0010CGQ, EZBioscience) for the complementary DNA synthesis. Quantification of the target gene expression was performed using the Color SYBR Green qPCR Master Mix (A0012‐R2, EZBioscience). Primer sequences applied for the gene amplification are listed in Table S5 (Supporting Information), with GAPDH serving as the housekeeping gene.

### Western Blot Analysis

Cells or tissues were lysed to isolate the total proteins with CelLytic M lysis buffer (Sigma‐Aldrich) or RIPA (Epizyme, Shanghai) containing both proteinase (Epizyme, Shanghai) and phosphatase inhibitors (Epizyme, Shanghai). For nuclear protein extraction, the nuclear protein extraction commercial kit (Beyotime, China) was applied, and the experimental procedures were carried out as per the manufacturers’ instructions. The extracted protein concentration was determined using a BCA protein assay kit (Beyotime, China).

Equal amounts of total proteins were electrophoresed in 10% or 12.5% w/v sodium dodecyl sulfate‐polyacrylamide gel electrophoresis (SDS‐PAGE) and electroblotted onto polyvinylidene fluoride (PVDF) membrane. After blocking by Protein‐free rapid blocking buffer (Epizyme) or 5% bovine serum albumin (BSA) according to the antibody supplier's recommendation, membranes were incubated with corresponding primary antibodies at 4 °C overnight. Then membranes were washed with Tris‐buffered saline‐tween (TBST) thrice and incubated with horseradish peroxidase‐conjugated secondary antibodies at room temperature for 1 h. The targeted protein bands were finally visualized using an enhanced chemiluminescence reagent (Epizyme, Shanghai) and photographed using the ChemiDoc CRS imaging system (Bio‐rad, USA). The primary antibodies used in this study are listed in Table S2 (Supporting Information).

### Coimmunoprecipitation (Co‐IP)

Lysate of TDSCs after different treatments were prepared using the IP Lysis/Wash buffer and subjected to the coimmunoprecipitation with Pierce Direct Magnetic IP/CO‐IP kit (#88 828, Thermo Fisher Scientific) according to the manufacturer's instruction. Briefly, anti‐HMGB1, anti‐TLR9 antibodies, or normal rabbit IgG were coupled with the magnet beads before incubation with the cell lysate on a rotator at 4 °C overnight. Then, the immunoprecipitants were eluted from the beads and mixed with 5 × loading buffer. The boiled lysates were subsequently loaded onto the 4–12% acrylamide gel and transferred onto the nitrocellulose membrane. Protein‐free rapid‐blocking buffer (Epizyme) was used for the blocking. After incubation, the membrane with the corresponding detecting antibodies overnight. Clean‐Blot IP detection reagent (#21 230, Thermo Fisher Scientific) was applied as a substitute for HRP‐conjugated secondary antibodies and incubated with the membrane the next day. Chemiluminescence was developed using the chemiluminescence reagent (Epizyme, Shanghai) and recorded on the ChemiDoc CRS imaging system (Bio‐rad, USA). The antibodies used are listed in Table S2 (Supporting Information).

### Luciferase Assay

Cells were seeded onto the 96‐well plate and cultured until 80% confluence. The NanoLuc reporter vector containing an NF‐*κ*B response element (NL3.2NF‐*κ*B‐RE reporter vector, #N1111, Promega, USA) was employed. Transfection was performed with the FuGENE HD transfection reagent (#E2311, Promega). After 48 h of transfection, cells were subjected to different treatments. The activity of NF‐*κ*B was assayed using the Nano‐Glo Luciferase Assay System (#N1110, Promega) according to the manufacturer's specification and calculated as the fold change of the control group (the fold of function).

### Enzyme‐Linked Immunosorbent Assay (ELISA)

Concentrations of cytokines in tissue homogenates, including TNF‐*α*, MCP‐1, IL‐6, and IL‐1*β* were measured by ELISA kits (Anogen, Canada) following the manufacturer's instructions. Cytokines, including CXCL‐1 and CXCL‐2 were determined using the ELISA kits (R&D system, USA) following the manufacturer's instructions. The total protein of samples was assayed using the BCA protein assay kit (Beyotime, China). Normalization was performed to the analyte concentration to be pg/µg of total protein.

### Statistical Analysis

Data are expressed as mean ± standard deviation (SD), and statistical analysis was performed by GraphPad Prism 9.0 (GraphPad Software Inc, USA). For analysis of the quantitative data, as to the comparison of two groups, unpaired two‐tailed Student's *t*‐test was conducted. As to the comparison of more than two groups, one‐way analysis of variance (ANOVA) with Tukey's multiple comparison tests as post hoc tests were performed. For the analysis of categorial data, the Chi‐square test was performed. Statistical significance was determined to be *p* value <0.05. PASS 15 software was used to compute the sample size for each experiment based on data from previous studies and preliminary experiments with an alpha level of 0.05% and 80% power. All experiments were carried out in at least three replicates.

## Conflict of Interest

The authors declare no conflict of interest.

## Author Contributions

J.L., X.W., Z.Y., and F.Y. contributed equally to this work. Research design, Conceptualization and funding acquisition: C.F., B.T., Z.S.; Study design and manuscript writing: J.L., X.W., Z.S., F.Y.; Experiment conduction: J.L., Z.Y., H.L.; Data collection, statistical analysis, and formal analysis: Z.S., Z.Y., G.L., Z.Y., X.Y., H.C. The authors read and approved the final manuscript.

## Supporting information

Supporting Information

## Data Availability

The data that support the findings of this study are available from the corresponding author upon reasonable request.
