# Supplementary material for: NLRP3‐Dependent Crosstalk between Pyroptotic Macrophage and Senescent Cell Orchestrates Trauma‐Induced Heterotopic Ossification During Aberrant Wound Healing
Source: Adv Sci (Weinh). 2023 May 19;10(19):2207383. doi: 10.1002/advs.202207383 (PMC10323626; doi:10.1002/advs.202207383)
Supplement: Supplementary file 1 — Supporting Information [file ADVS-10-2207383-s001.pdf]

ADVANCED SCIENCE
Open Access

Supporting Information

for *Adv. Sci.*, DOI 10.1002/advs.202207383

NLRP3-Dependent Crosstalk between Pyroptotic Macrophage and Senescent Cell Orchestrates Trauma-Induced Heterotopic Ossification During Aberrant Wound Healing

Juehong Li, Xin Wang, Zhixiao Yao, Feng Yuan, Hang Liu, Zhenyu Sun, Zhengqiang Yuan, Gang Luo, Xiangyun Yao, Haomin Cui, Bing Tu, Ziyang Sun* and Cunyi Fan**

Supplementary Materials for

NLRP3-dependent crosstalk between pyroptotic macrophage and senescent cell orchestrates trauma-induced heterotopic ossification during aberrant wound healing

Juehong Li *et al.*

*Corresponding authors: Bing Tu (tubing216@163.com)
Ziyang Sun (sternhimmel96@163.com)
Cunyi Fan (cyfan@sjtu.edu.cn)

This PDF file includes:

Figs. S1 to S14
Tables S1 to S5

Supplementary materials and methods

CCK-8 assay

To ensure concentrations of Quercetin (Selleck, USA) we used were non-cytotoxic, Cell Counting Kit-8 assay was conducted. After inoculated onto a 96-well plate at a density of 6×10^3 cells/well in triplicate, TDSCs were incubated in complete α–MEM supplemented with 100 μM hydrogen peroxide (H2O2) with or without 20 μM Quercetin for 1, 3 and 7 days. The medium was refreshed every 2 days. At the end of experiments, 10 ul CCK-8 solution (Dojindo, Kumamoto, Japan) was added to each well and incubated at 37°C for 2 hours. The optical density (OD) value was determined using SpectraMax i3x (Molecular Devices, Australia) at an absorbance of 450 nm.

In vivo tracing of the CM-Dil-labeled EVs

EVs were labeled with 1 μM CM-Dil (Molecular Probes, USA) using the same method as that described in Dil. After labeling EVs with CM-Dil, CM-Dil-labeled EVs were locally administrated into the injury site (20 ug in 10 ul PBS) of Achilles tendon upon time of surgery, 1 week after surgery, the mice were sacrificed and the regenerated tendon from the tendomuscular junction to the enthesis were excised and fixed. Routine dehydration, paraffin embedding and sectioning was performed. Immunofluorescent staining for PDGFRα and p16 was conducted. DAPI solution was used for nucleus staining. The primary antibodies used in are listed in Supplementary Table 2.

Rescue assay for senescent TDSCs

TDSCs were seeded onto the 24-well plate and were induced to senescence by 100 μM hydrogen peroxide (H2O2) for 24 hours. Quercetin at 20 μM or vehicle was then applied for senescent TDSCs rejuvenation for 48 hours. The cellular senescence of TDSCs was detected by Senescence associated β-galactosidase (SA-β-Gal) staining. After that, osteogenic induction was performed using the osteogenic differentiation medium (Cyagen, China). At 7 days, Alkaline Phosphatase (ALP) staining was performed using the BCIP/NBT Alkaline Phosphatase Color Development Kit (Beyotime, China) and ALP activity was examined using the alkaline phosphatase assay kit (Nanjing Jiancheng Biotechnology Co Ltd, China). At 21 days, alizarin red S (ARS) staining was performed with 2% Alizarin Red S (pH 4.2) solution. 10% cetylpyridine chloride was applied for ARS dye solvation and quantification by detecting the absorbance at 570 nm.

Synergistic effects of IL-1β on the senescence and osteogenesis of TDSCs

To examine the synergistic effects of IL-1β with other pro-inflammatory cytokines on the senescence and osteogenesis of TDSCs, TDSCs were treated with 10 ng/ml TGF-β, 2 ng/ml IL-6 or 10 ng/ml TNF-α respectively in the presence or absence of 10 ng/ml IL-1β for 24 hours. SA-β-Gal staining was performed for cellular senescence visualization. For osteogenic potential detection, osteogenic induction of TDSCs was performed with the osteogenic differentiation medium (Cyagen, China) supplemented with 10 ng/ml TGF-β, 2 ng/ml IL-6 or 10 ng/ml TNF-α respectively and combined with 10 ng/ml IL-1β or not. ALP staining and ALP activity were determined at 7 days. ARS staining and quantification was conducted at 21 days.

Fig. S1.

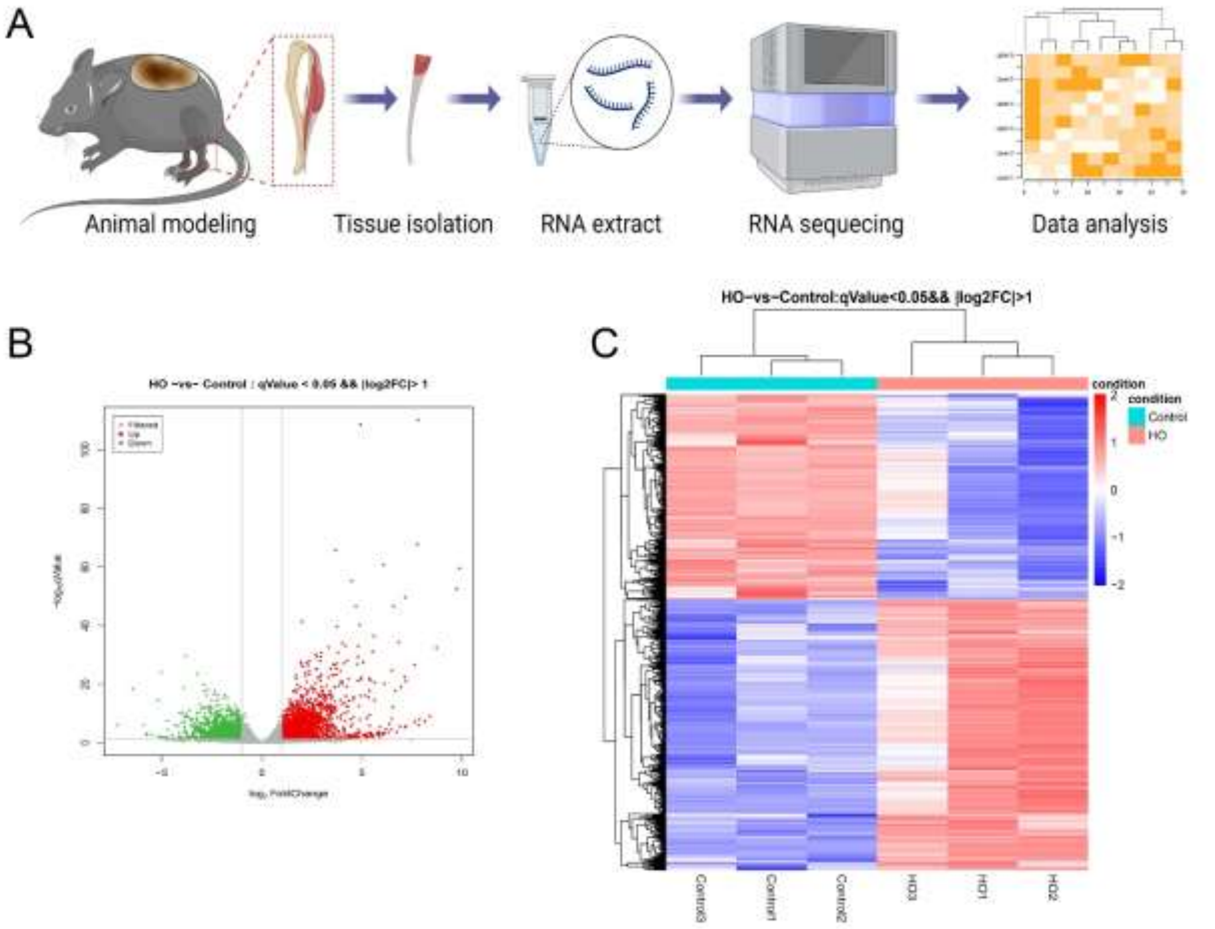

Supplementary figure 1. RNA sequencing of HO-forming regenerative tendon after injury.

(A) Work flow for tissue harvest and RNA sequencing; (B) Volcano plot for differentially expressed genes (DEG) between normal tendon (Sham group, Control) and HO-forming regenerative tendon (HO group, HO); (C) Heatmap of DEG between tendon in Sham (Control) and HO groups. N=3/group.

Fig. S2.

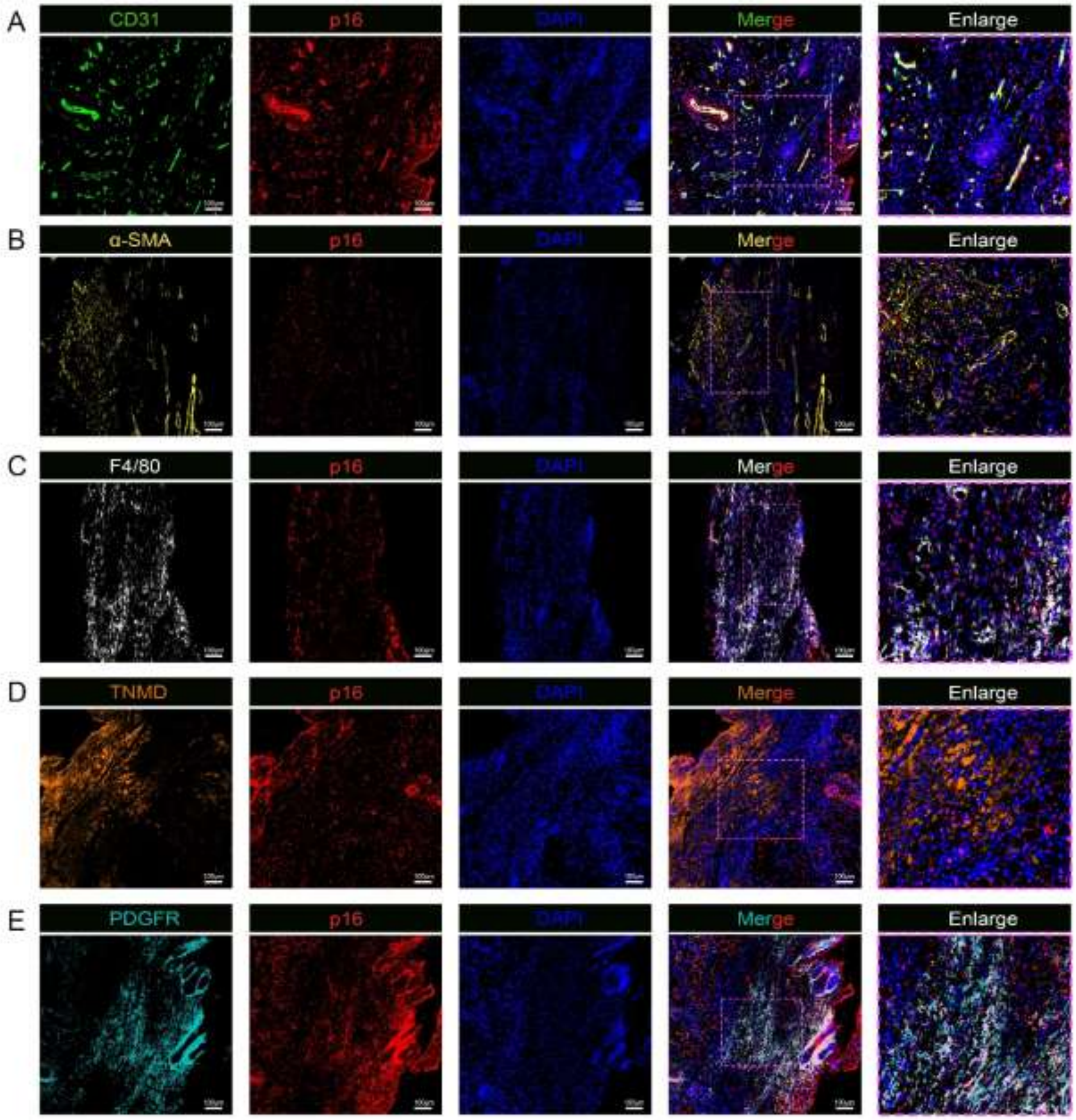

Supplementary figure 2. Identification of cellular origin of p16+ senescent cells within regenerative tendon tissues.

(A) Double immunofluorescence staining of senescent marker p16 and endothelial marker CD31 for co-localization analysis. (B) Double immunofluorescence staining of senescent marker p16

and fibroblast marker α-SMA for co-localization analysis. (C) Double immunofluorescence staining of senescent marker p16 and macrophage marker F4/80 for co-localization analysis. (D) Double immunofluorescence staining of senescent marker p16 and tenocyte marker TNMD for co-localization analysis. (E) Double immunofluorescence staining of senescent marker p16 and osteoprogenitor marker PDGFRα for co-localization analysis. Images labeled Enlarge were magnified images of the boxed areas in the images labeled Merge. N=6/group.

Fig. S3.

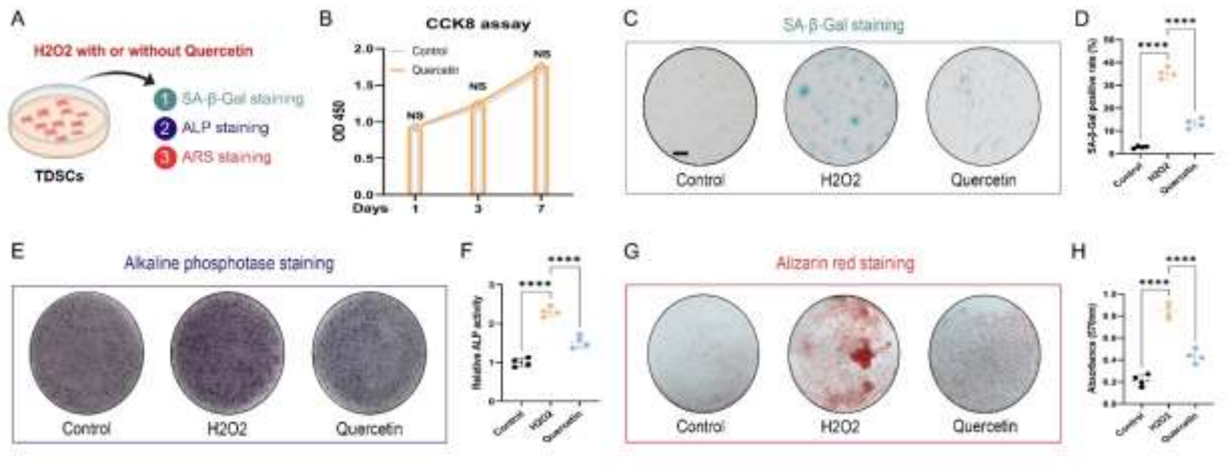

Supplementary figure 3. Senolytic treatment abrogated the elevated osteogenic potential of senescent TDSCs.

(A) Illustration of the in vitro experimental treatment design. (B) CCK8 assay of TDSCs treated with quercetin for 1, 3 and 7 days. (C) SA-β-Gal staining for TDSCs after indicated treatments. (D) Quantification of SA-β-Gal positive rate. (E) ALP staining for TDSCs following indicated treatments at 7 days after osteogenic induction. (F) Quantification of ALP activity for TDSCs following indicated treatments at 7 days after osteogenic induction. (G) ARS staining for TDSCs following indicated treatments at 21 days after osteogenic induction. (H) Quantification of absorbance of eluted ARS staining at 570 nm. N=4/group. Data are presented as mean ± SD. Unpaired two-tailed student's t-test for panel B. One-way ANOVA followed by Tukey's test for panel C-H. ****$P < 0.0001$ and NS: not significant.

Fig. S4.

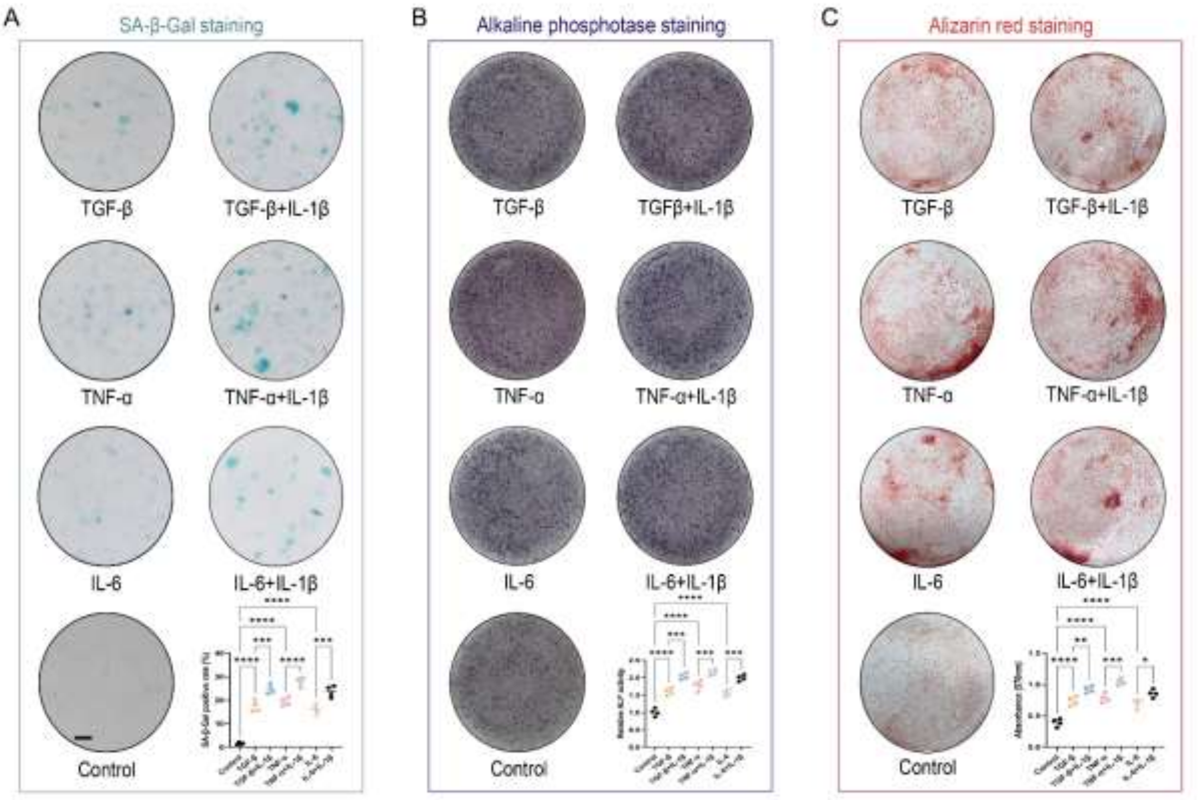

Supplementary figure 4. The synergic effects of IL-1β with other pro-inflammatory cytokines on senescent and osteogenic changes of TDSCs.

(D) ALP staining for TDSCs following indicated treatments at 7 days after osteogenic induction. (E) Quantification of ALP activity for TDSCs following indicated treatments at 7 days after osteogenic induction. (F) ARS staining for TDSCs following indicated treatments at 21 days after osteogenic induction. (G) Quantification of absorbance of eluted ARS staining at 570 nm. N=4/group. Data are presented as mean ± SD. One-way ANOVA followed by Tukey’s test. *$P < 0.05$, **$P < 0.01$, ***$P < 0.001$, ****$P < 0.0001$.

Fig. S5.

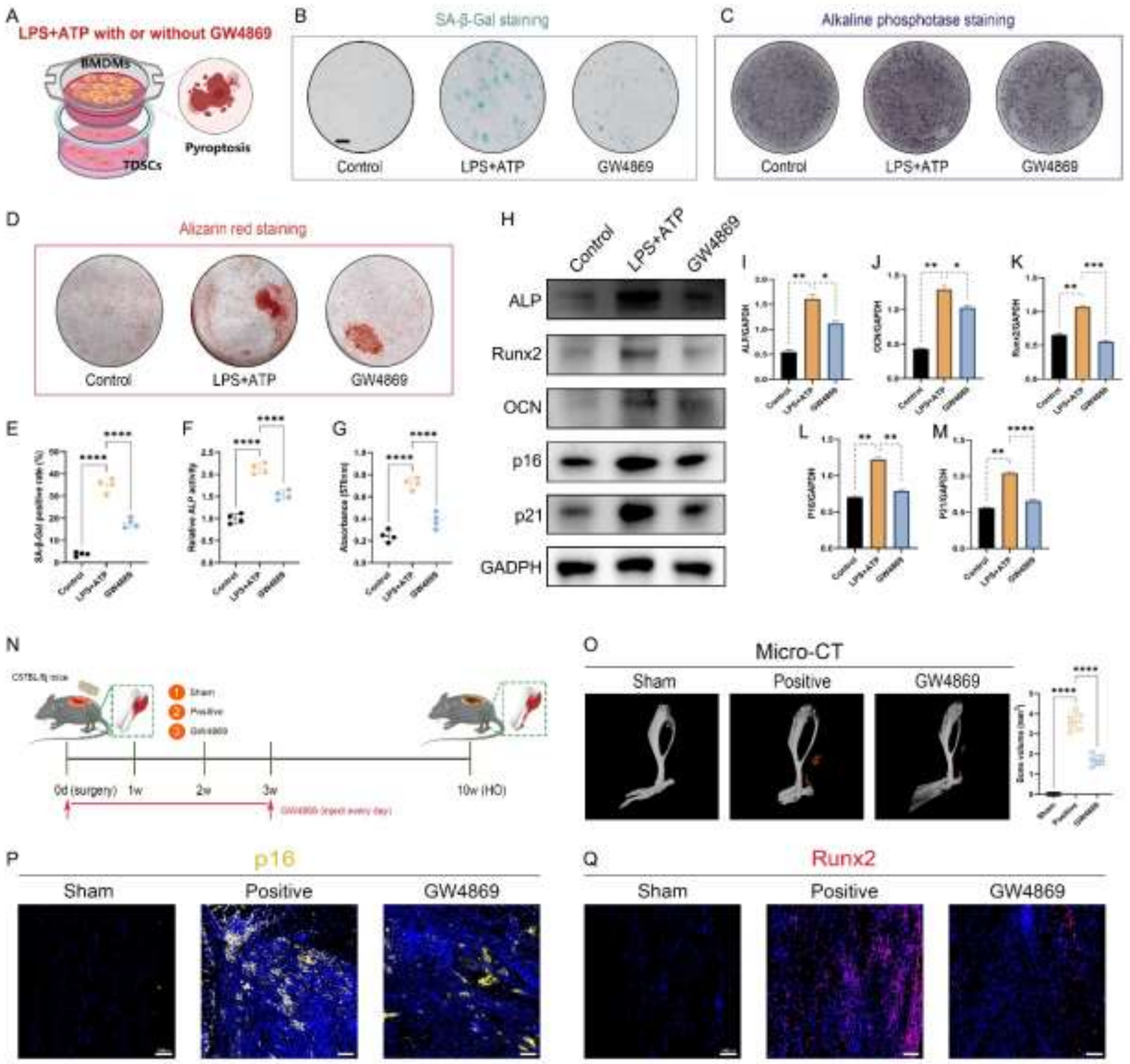

Supplementary figure 5. EVs secretion abrogation reversed the pro-senescent and pro-osteogenic effects of pyroptotic macrophages on TDSCs during trauma-induced HO formation.

(A) Illustration of the in vitro coculture system and experimental treatment design; (B) SA-β-Gal staining for TDSCs after indicated treatments. (C) ALP staining for TDSCs following indicated treatments at 7 days after osteogenic induction. (D) ARS staining for TDSCs following indicated

treatments at 21 days after osteogenic induction. (E) Quantification of SA-β-Gal positive rate. (F) Quantification of ALP activity for TDSCs following indicated treatments at 7 days after osteogenic induction. (G) Quantification of absorbance of eluted ARS staining at 570 nm. N=4/group. (H) WB analysis for cellular senescence marker p16, p21 and osteogenic marker Runx2, ALP and OCN. (I-M) Quantification of protein level of p16, p21, Runx2, ALP and OCN normalized to GAPDH. N=3/group. (N) Illustration of the experimental design in the murine burn/tenotomy model. (O-P) Micro-CT analysis and quantification for HO formation following indicated treatments at 10 weeks after injury. (Q) Immunofluorescent staining of p16 in regenerative tendon tissues following indicated treatments at 7 days after injury. (R) Immunofluorescent staining of Runx2 in regenerative tendon tissues following indicated treatments at 7 days after injury. N=6/group. Data are presented as mean±SD. One-way ANOVA followed by Tukey's test. *$P < 0.05$, **$P < 0.01$, ***$P < 0.001$, and ****$P < 0.0001$.

Fig. S6.

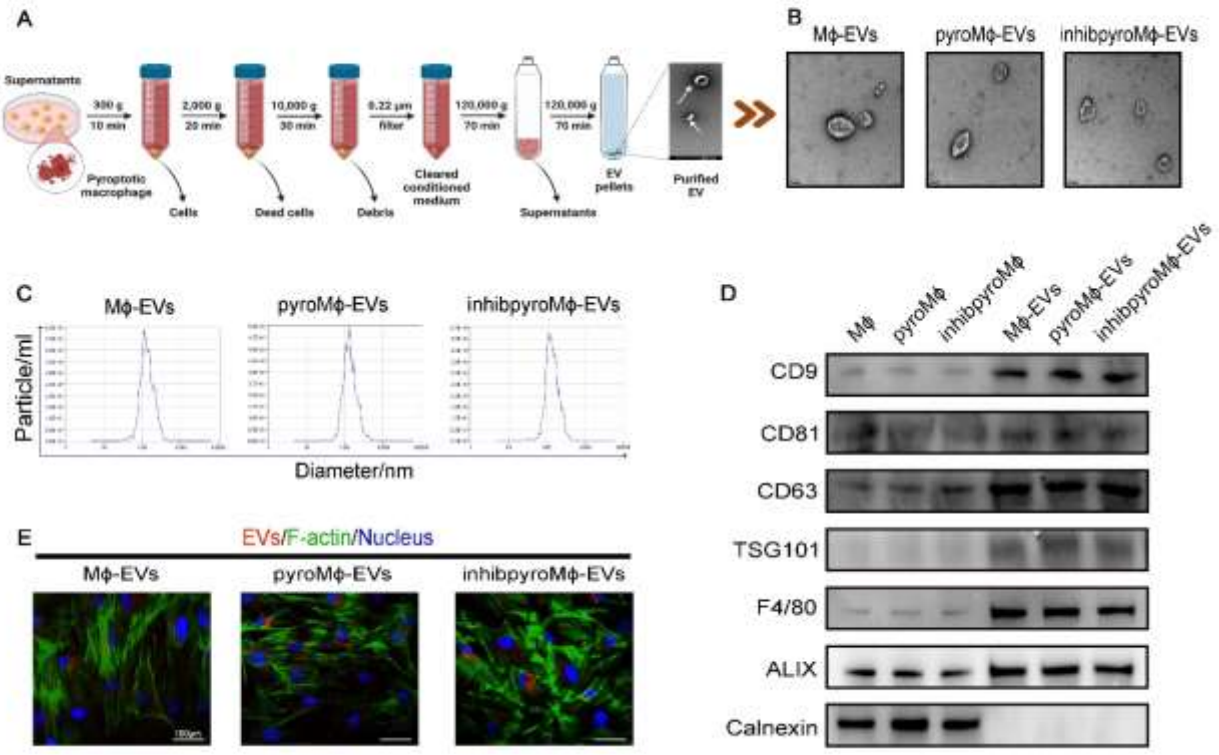

Supplementary figure 6. Isolation and characterization of EVs from macrophages under different conditions

(A) Work flow for isolation of EVs from supernatants of wild type or NLRP3-/- macrophages upon pyroptosis induction or not. (B) Transmission electron microscopic images for EVs observation. (C) nanoparticle tracking analysis for particle size determination of EVs. (D) WB analysis for identification of the EVs positive markers including CD81, CD9, CD63, TSG101, ALIX, negative marker Calnexin and parental cell marker F4/80. (E) Fluorescent images for checking of EVs internalization in TDSCs. Red indicated Dil-labeled EVs, green indicated iFluor 488-labeled phalloidin, Blue indicated DAPI. N=3/group.

Fig. S7.

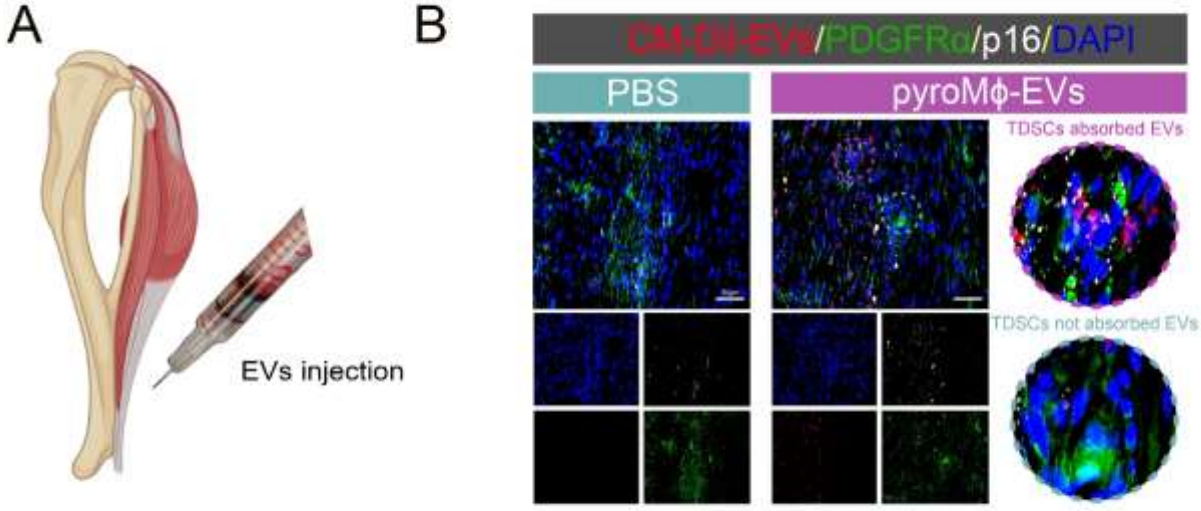

Supplementary figure 7. In vivo tracing of the fate and pro-senescent effects of EVs from pyroptotic macrophages.

(A) Illustration of the injection of CM-Dil-labeled EVs in regenerative tendon. (B) Representative images of distribution of CM-Dil-labeled EVs and co-localization with the tendon stem cells marker PDGFRα and senescent marker p16 in regenerative tendon. N=3/group.

Fig. S8.

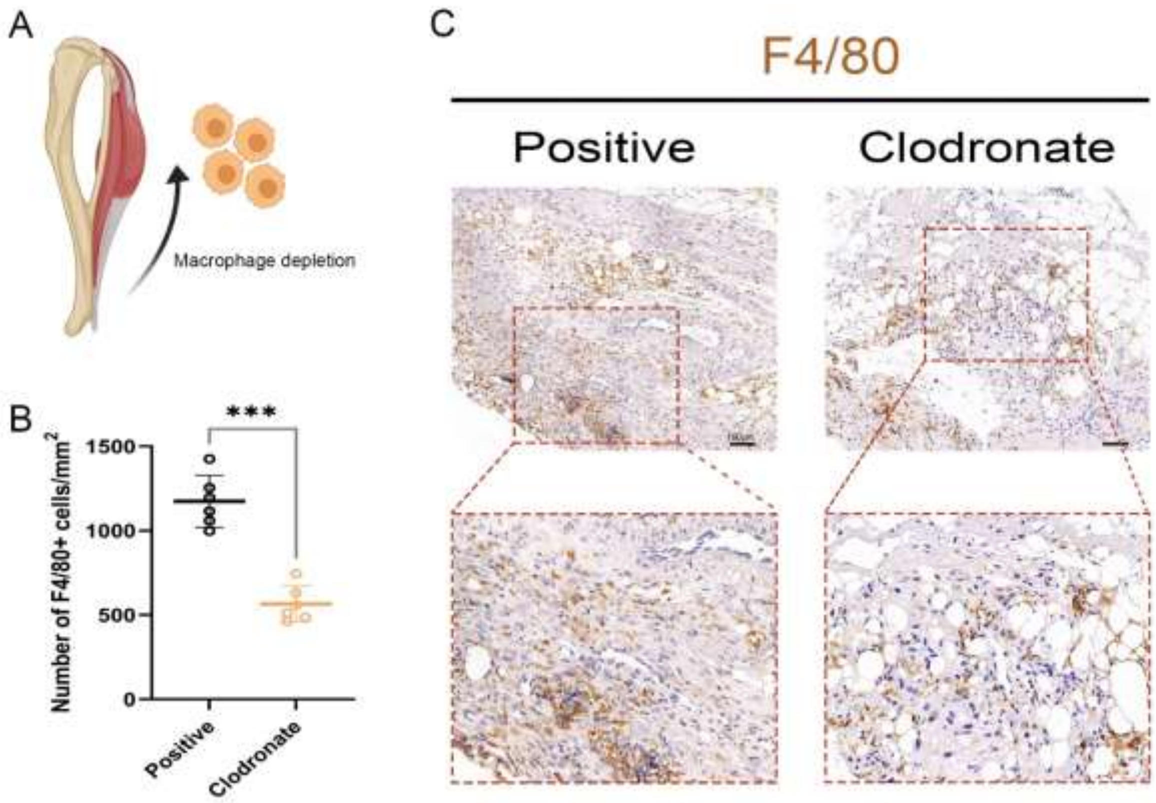

Supplementary figure 8. Depletion of macrophages in regenerative tendon using clodronate liposome.

(A) Illustration of the macrophage depletion by clodronate liposome in regenerative tendon. (B, C) Immunohistochemical staining and quantification of F4/80 for determination of the macrophage infiltration in the regenerative tendon. N=6/group. Data are presented as mean±SD. Unpaired two-tailed student's t-test. ****P*＜0.001.

Fig. S9.

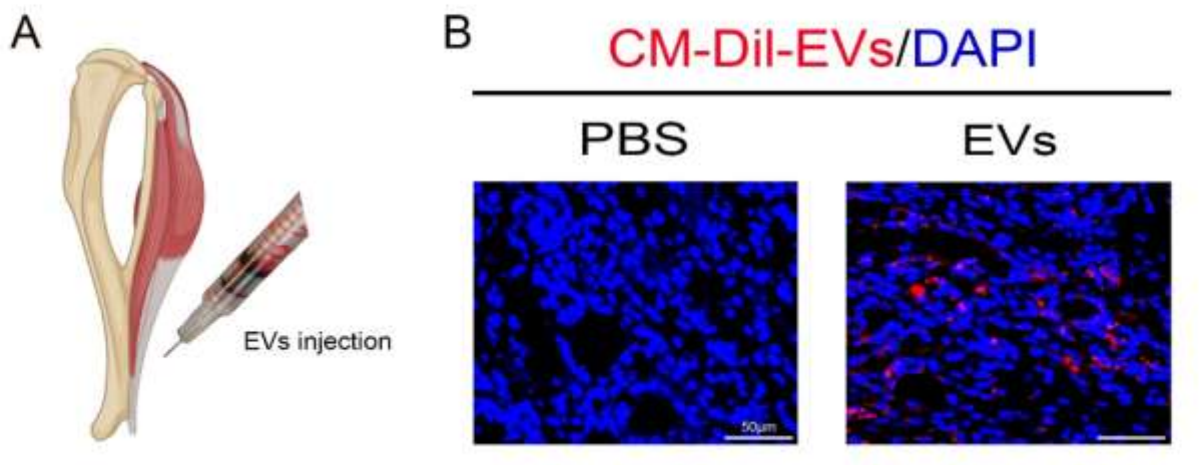

Supplementary figure 9. Uptake of locally injected EVs in regenerative tendon.

(A) Illustration of the injection of Dil-labeled EVs in regenerative tendon. (B) Representative images of uptake of Dil-labeled EVs in regenerative tendon. N=3/group.

Fig. S10.

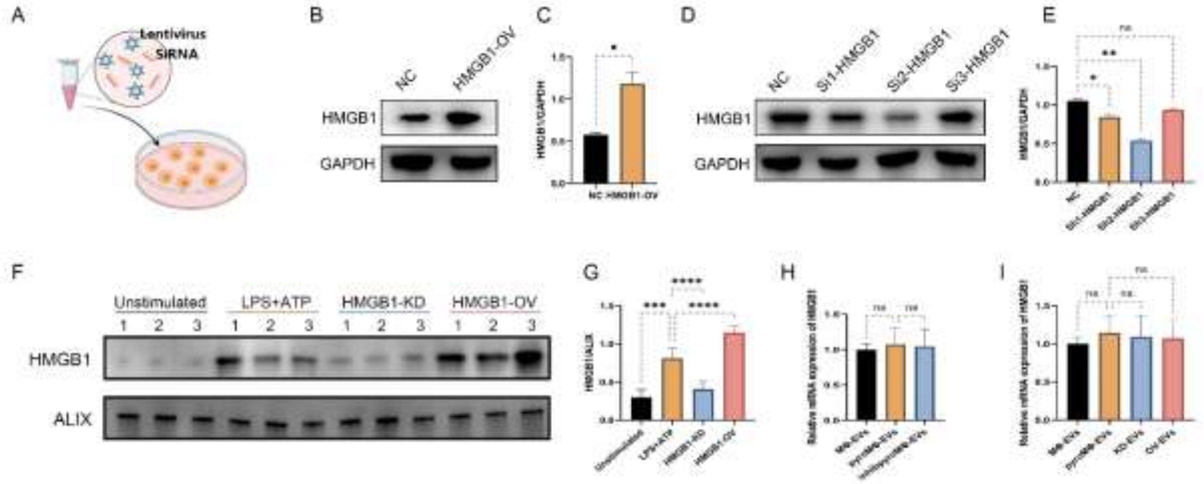

Supplementary figure 10. Transfection efficiency of knockdown and overexpression of HMGB1 in BMDM.

(A) Illustration of the transfection in BMDMs. (B, C) The WB analysis for HMGB1 overexpression (HMGB1-OV) in BMDMs with lentivirus. (D, E) The WB analysis for HMGB1 knockdown in BMDMs with three candidate siRNA (Si-HMGB1). (F, G) The WB analysis for HMGB1 incorporation in EVs isolated from BMDMs after indicated treatments. (H, I) The qRT-PCR analysis for HMGB1 mRNA expression in TDSCs after indicated EVs treatments.

N=3/group. Data are presented as mean $\pm$ SD. One-way ANOVA followed by Tukey's test (E, G-I) and unpaired two-tailed student's t-test (C). *$P<0.05$, **$P<0.01$, ***$P<0.001$, ****$P<0.0001$ and NS: not significant.

Fig. S11.

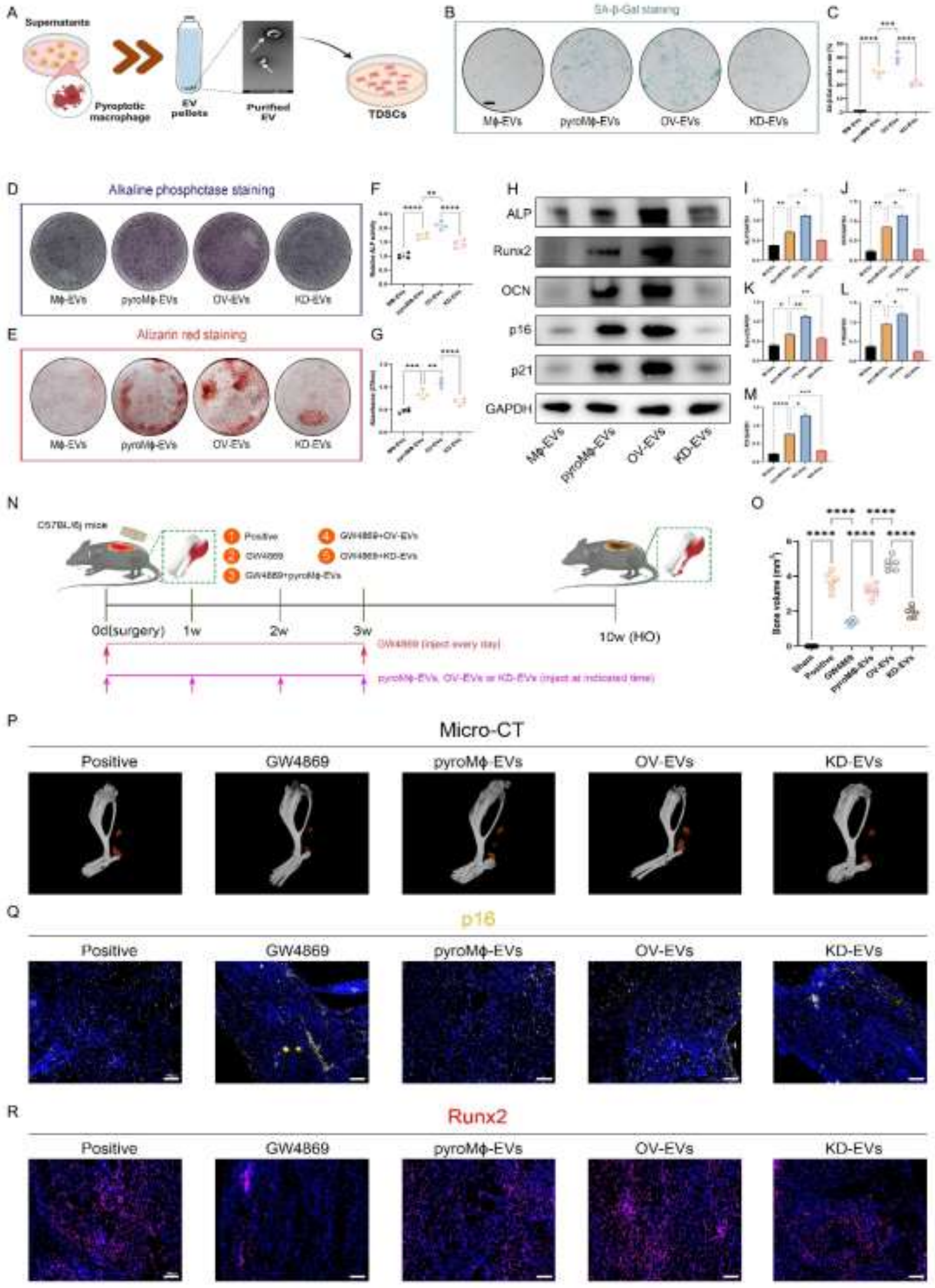

Supplementary figure 11. HMGB1 was in charge of the undesirable effects of EVs from pyroptotic macrophages on TDSCs during trauma-induced HO formation.

(A) Illustration of the in vitro coculture system and experimental treatment design. (B) SA-β-Gal staining for TDSCs after indicated treatments. (C) Quantification of SA-β-Gal positive rate. (D) ALP staining for TDSCs following indicated treatments at 7 days after osteogenic induction. (E) Quantification of ALP activity for TDSCs following indicated treatments at 7 days after osteogenic induction. (F) ARS staining for TDSCs following indicated treatments at 21 days after osteogenic induction. (G) Quantification of absorbance of eluted ARS staining at 570 nm. N=4/group. (H) WB analysis for cellular senescence marker p16, p21 and osteogenic marker Runx2, ALP and OCN. (I-M) Quantification of protein level of p16, p21, Runx2, ALP and OCN normalized to GAPDH. N=3/group. (N) Illustration of the experimental design in the murine burn/tenotomy model. (O-P) Micro-CT analysis and quantification for HO formation following indicated treatments at 10 weeks after injury. (Q) Immunofluorescent staining of p16 in regenerative tendon tissues following indicated treatments at 7 days after injury. (R) Immunofluorescent staining of Runx2 in regenerative tendon tissues following indicated treatments at 7 days after injury. N=6/group. Data are presented as mean ± SD. One-way ANOVA followed by Tukey's test. *$P < 0.05$, **$P < 0.01$, ***$P < 0.001$ and ****$P < 0.0001$.

Fig. S12.

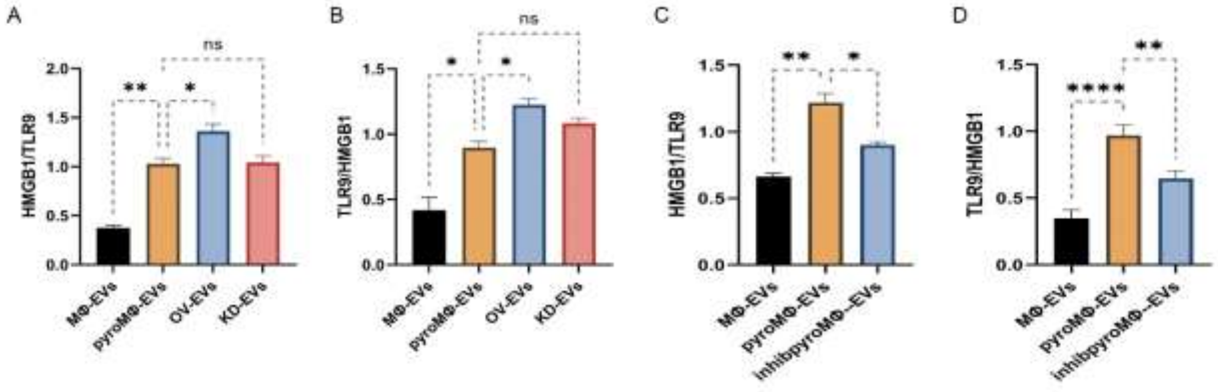

Supplementary figure 12. Quantitative analysis for co-immunoprecipitation assay.

(A, C) Relative protein level of HMGB1 normalized to TLR9. (B, D) Relative protein level of TLR9 normalized to HMGB1. N=3/group. Data are presented as mean ± SD. One-way ANOVA followed by Tukey's test. $*P < 0.05$, $**P < 0.01$, $****P < 0.0001$ and NS: not significant.

Fig. S13.

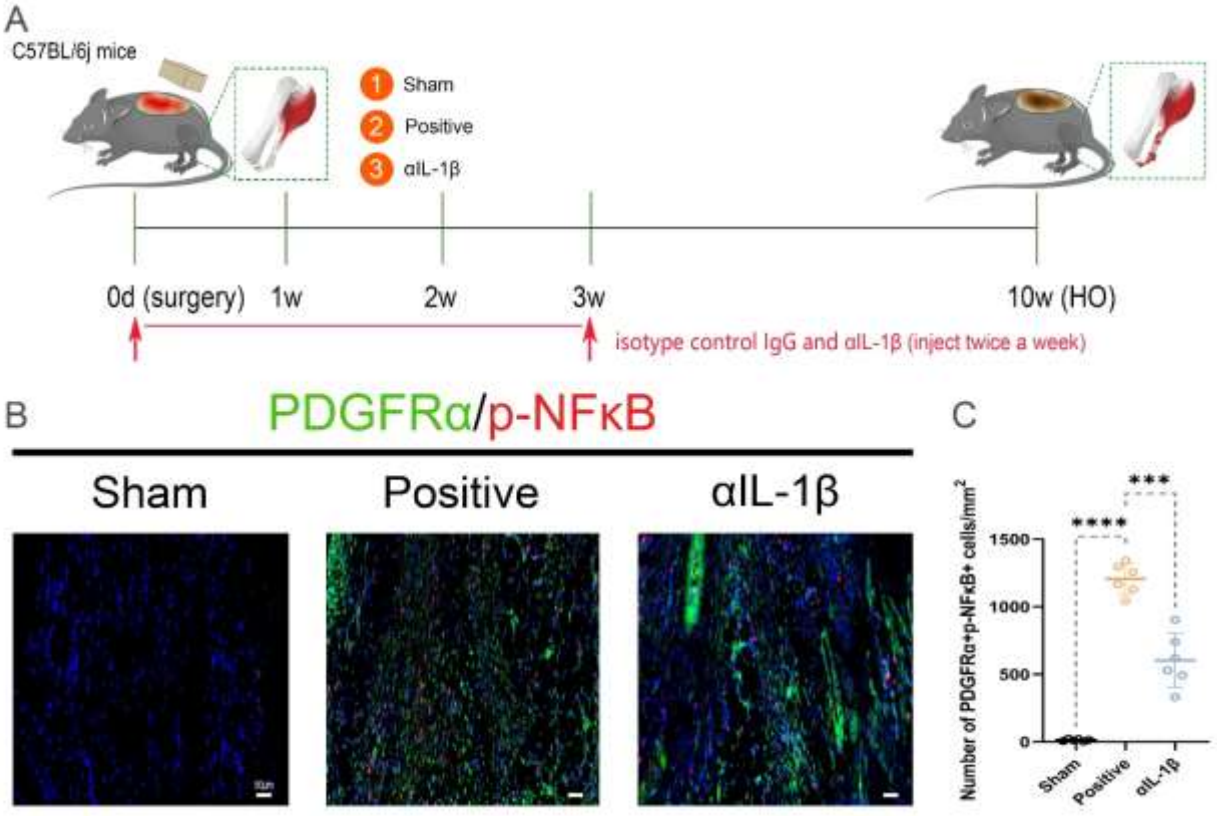

Supplementary figure 13. Neutralization of IL-1β abrogated NF-κB signaling in osteoprogenitors within the murine traumatic HO model.

(A) Illustration of in vivo experimental protocols, (B, C) Analysis and quantification of PDGFRα+p-NF-κB p65 cells in the regenerative tendon tissues by double immunofluorescence staining at 7 days after indicated treatment. N=6/group. Data are presented as mean±SD. One-way ANOVA followed by Tukey's test. ***$P < 0.001$, ****$P < 0.0001$.

Fig. S14.

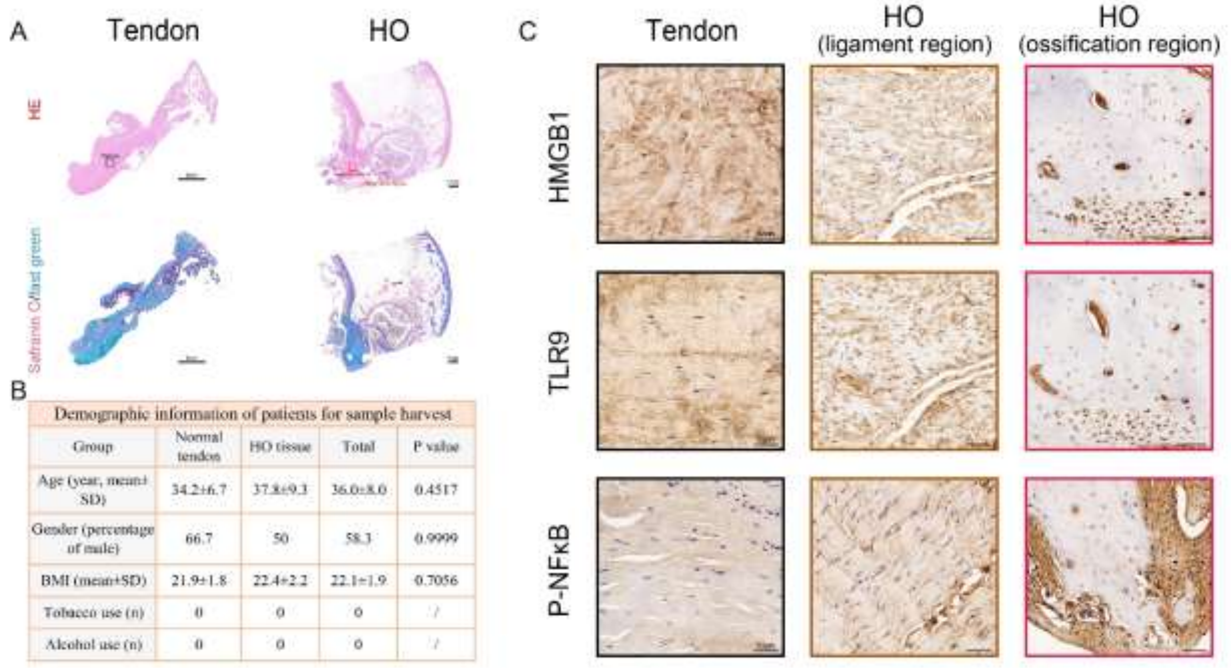

Demographic information of patients for sample harvest				
Group	Normal tendon	HO tissue	Total	P value
Age (year, mean±SD)	34.2±6.7	37.8±9.3	36.0±8.0	0.4517
Gender (percentage of male)	66.7	50	58.3	0.9999
BMI (mean±SD)	21.9±1.8	22.4±2.2	22.1±1.9	0.7056
Tobacco use (n)	0	0	0	/
Alcohol use (n)	0	0	0	/

Supplementary figure 14. Activation of HMGB1/TLR9/NF-κB signaling in clinical HO samples.

(A) HE staining and Masson's trichrome staining for the HO tissue section and normal tendon tissue section. Black rectangular box defined the normal tendon region in the normal tendon tissue section, brown rectangular box defined the ligament region in the HO tissue section, red rectangular box defined the ossification region in the HO tissue section; (B) Demographical information of enrolled patients for clinical sample collection; (C) Immunohistochemical staining for HMGB1, TLR9 and p-NF-κB in the HO tissue section and normal tendon tissue section. N=6/group. Data are presented as mean ± SD. Unpaired two-tailed student's t-test and chi-square test.

Table S1. Sequences used for RNAi

Tools	Sense	Anti-sense
HMGB1 siRNA-1	GGCUGACAAGGCUCGUUAU	AUAACGAGCCUUGUCAGCC
HMGB1 siRNA-2	UGCUGCCUACAGAGCUAAA	UUUAGCUCUGUAGGCAGCA
HMGB1 siRNA-3	CCUGUCCAUUGGUGAUGUUTT	AACAUCACCAAUGGACAGGTT

Table S2. Antibodies used in this study

Antibodies	Supplier	Catalog	Application
NLRP3	Proteintech	19771-1-AP	WB, IF
Caspase 1	Proteintech	22915-1-AP	WB
Cleaved-Caspase 1	Affinity	AF4022	IF
Cleaved-IL-1β	Affinity	AF4006	IHC
IL-1β	BioXCell	BE0246	Neutralization
HMGB1	Proteintech	10829-1-AP	WB, IP, IF
HMGB1	Proteintech	66526-1-lg	IF
TLR9	Novus biologicals	NBP2-24729	WB, IF, IP
NFκB p65	Cell signaling technology	8242	WB, IF
Phospho-NFκB p65 (Ser536)	Cell signaling technology	3033	WB
EEA1	Proteintech	28347-1-AP	IF
F4/80	Cell signaling technology	70076	IHC, IF
α-SMA	Proteintech	14395-1-AP	IF
CD31	Abcam	ab28364	IF
TNMD	Abcam	ab203676	IF
PDGFRα	Cell signaling technology	3174	IF
CD9	Proteintech	60232-1-lg	WB
CD81	Proteintech	27855-1-AP	WB
CD63	Proteintech	25682-1-AP	WB
CD63	Abcam	ab217345	IF

**TSG101**	**Proteintech**	**28283-1-AP**	**WB**
**Calnexin**	**Proteintech**	**10427-2-AP**	**WB**
**Alix**	**Proteintech**	**12422-1-AP**	**WB**
**P16**	**Proteintech**	**10883-1-AP**	**WB, IF**
**P21**	**Proteintech**	**10355-1-AP**	**WB**
**ALP**	**Abcam**	**ab95462**	**WB**
**Runx2**	**Proteintech**	**20700-1-AP**	**WB**
**OCN**	**Abcam**	**ab93876**	**WB**
**OPN**	**Proteintech**	**22952-1-AP**	**IHC**
**Histone H3**	**Affinity**	**AF0863**	**WB**
**GAPDH**	**Proteintech**	**60004-1-lg**	**WB**

Table S3. Primer sequence used for genotyping NLRP3 knockout mice

Primer identifier	Sequence (5'→3')	Primer type
16568	TGCCTGGTCTTTACTGAAGG	Mutant
16703	TCAGTTTCCTTGGCTACCAGA	Wild type
16704	TTCCATTACAGTCACTCCAGATGT	Common

Table S4. Reagents and chemicals used in this study

Reagents	Supplier	Catalog
GW4869	Selleck	S7609
JSH-23	Selleck	S7351
Quercetin	Selleck	S2391
AT791	MedChemExpress	HY-124603
Clodronate liposomes	Liposoma B. V.	CP-005-005
Polyclonal armenian hamster IgG	BioXCell	BE0091
Murine recombinant IL-1β	Peprotech	211-11B
Murine recombinant TNF-α	Peprotech	315-01A
Mouse recombinant TGFβ1	R & D system	7666-MB

Table S5. Primers used for qRT-PCR

Genes	Forward	Reverse
Murine HMGB1	CCAAGAAGTGCTCAGAGAGGTG	GTCCTTGAACTTCTTTTTGGTCTC
Murine CXCL1	TCCAGAGCTTGAAGGTGTTGCC	AACCAAGGGAGCTTCAGGGTCA
Murine CXCL2	CATCCAGAGCTTGAGTGTGACG	GGCTTCAGGGTCAAGGCAAACT
Murine TNF-α	TAGCCAGGAGGGAGA ACAGA	CCAGTGAGTGAAAGGGACAGA
Murine MCP-1	CATCCACTACCTTTTCCACAA	CATCACAGTCCGAGTCACAC
Murine IL-6	ACCAAGACCATCCAATTCATC	CTGACCACAGTGAGGAATGTC
Murine GAPDH	CCTCGTCCCGTAGACAAAATG	TGAGGTCAATGAAGGGGTCGT
